# Association Between Gall Structural and Metabolic Complexity: Evidence from *Pistacia palaestina*

**DOI:** 10.3390/plants14050721

**Published:** 2025-02-26

**Authors:** Daniela Batovska, Mirena Chakarova, Monica Dines, Ivayla Dincheva, Ilian Badjakov, Moshe Inbar

**Affiliations:** 1 Institute of Chemical Engineering, Bulgarian Academy of Sciences, Acad. G. Bonchev Str., Bl. 103, 1113 Sofia, Bulgaria; dmirena@abv.bg; 2 Department of Evolutionary and Environmental Biology, University of Haifa, Haifa 3498838, Israel; monica.dines@gmail.com; 3 Department of Agrobiotechnologies, Agrobioinstitute, Agricultural Academy, 8 Dragan Tsankov Blvd., 1164 Sofia, Bulgaria; ivadincheva@abi.bg (I.D.); ibadjakov@abi.bg (I.B.)

**Keywords:** aphids, *Baizongia*, gall complexity, GC–MS, *Geoica*, host plant manipulation, metabolomics, *Paracletus*

## Abstract

*Pistacia palaestina* hosts several Fordini gall-forming aphid species, which manipulate its anatomy and metabolism, creating galls that provide nutrients and protection. This study compared the extended metabolic profiles of *P. palaestina* leaves and galls induced by *Baizongia pistaciae*, *Paracletus cimiciformis*, and *Geoica* spp. GC–MS analysis of ethyl acetate (EtOAc) and methanol (MeOH) extracts revealed a high abundance of shikimic acid and quinic acid isomers, along with diverse hydrocarbons, lipids, terpenoids, phenolics, and carbohydrates, each showing distinct distributions across gall types. *Paracletus cimiciformis* galls closely resembled intact leaves, exhibiting limited metabolic disruption. In contrast, the larger, more complex galls of *Baizongia* and *Geoica* underwent profound metabolic modifications. These aphids manipulate host metabolism, leading to triterpenoid and phenolics accumulation, which likely fortifies gall structure and enhances chemical defense. The considerable variation among individual trees suggests that specific host plant templates significantly influence the metabolic profile of the galls.

## 1. Introduction

This study focuses on *Pistacia palaestina* Boiss., a subspecies of the European *P. terebinthus* in the Mediterranean region. The tree serves as an obligate host for approximately eight species of galling aphids (Hemiptera: Aphididae, Fordini) [1,2]. These phloem-feeding specialists induce galls on young, unfolding leaves in spring, with each aphid species forming its own characteristic type of gall. Within the galls, the aphids gain shelter and nutrients while manipulating the host tree’s metabolism, physiology, and anatomy to create a favorable environment for their growth and development.

Although the mechanism of gall formation by insects is not fully understood, it is most probably triggered and controlled by plant growth regulators and signaling molecules secreted by aphids [3,4]. These interactions highlight the complex physiological and molecular relationship between aphids and their hosts [5].

It has been shown that Fordini-induced galls on *Pistacia* have higher levels of tannins, volatile terpenoids (mainly mono- and sesquiterpenoids), triterpenoids, and total terpenoids compared to intact leaves [6,7,8,9]. The metabolic variation exists at multiple levels—within individual trees, among different trees, and across aphid species [6,10]. Monoterpenes appear to be synthesized in situ in gall tissues, with upregulation of genes in terpene biosynthetic pathways observed in *P. palaestina* [11]. For example, the large galls of *B. pistaciae* are particularly enriched with monoterpenes, including α-thujene, sabinene, camphene, α-pinene, β-myrcene, δ-3-carene, terpene-4-ol, and γ-terpinene, whereas sesquiterpenes are more prevalent in leaves [6,7,9,12]. These phytochemicals deter predators, parasitoids, and herbivores [6,13,14] and exhibit antipathogenic properties [15].

While the essential oil composition of *P. palaestina* galls, induced by *B. pistaciae*, has been extensively studied, the broader metabolic profile of both galls and leaves, particularly in response to aphid-induced changes, remains underexplored. Due to their unique physiological and ecological roles, galls are recognized as rich reservoirs of bioactive compounds, concentrating secondary metabolites such as phenolics, terpenoids, alkaloids, and lipophilic components [16]. Such metabolites not only enhance the galls’ defense mechanisms but also represent a valuable source of bioactive molecules with potential pharmacological applications [17]. Galls also have a history of use in traditional medicine [16]. Therefore, revealing the broad range of metabolites may lead to practical usage.

For the first time, this study characterizes, quantifies, and compares the large-scale metabolite profiles of leaves and three distinct gall types induced by aphids on wild *P. palaestina* trees using an untargeted metabolomics approach with GC–MS. This method was chosen for its ability to comprehensively analyze and compare a wide range of metabolites without prior assumptions, providing valuable insights into the metabolic profiles of both leaf and gall tissues.

**Figure 1:**
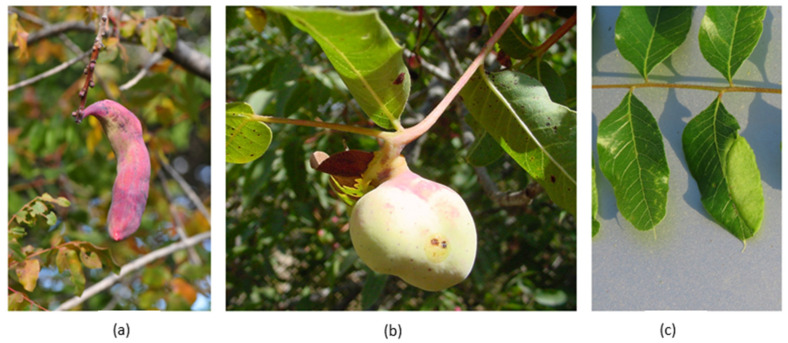
Galls induced on *P. palaestina* by *B. pistaciae* (**a**), *Geoica* spp. (**b**), and *P. cimiciformis* (**c**).

Plant material was collected from three naturally growing trees in Israel and sequentially extracted with EtOAc and MeOH to obtain compounds with varying polarity. We collected and analyzed *P. palaestina* leaves and galls induced by three aphid species that differ in their structural complexity [5]:

1. Banana-like (in shape and size) galls induced by *B. pistaciae*, which can house thou-sands of aphids and significantly impact the entire shoot (Figure 1a).
2. Spherical galls induced by *Geoica* spp. on leaflet midribs, supporting hundreds of aphids (Figure 1b).
3. Flat, open galls induced by *P. cimiciformis* on leaflet margins, with minimal impact on the plant and housing fewer than 100 aphids (Figure 1c).

The primary objective was to elucidate *P. palaestina*’s metabolic response to different aphid species by exploring the differential accumulation of bioactive compounds between leaves and galls.

## 2. Results

### 2.1. Metabolic Profiles of EtOAc Extracts from Galls and Leaves

**Figure 2:**
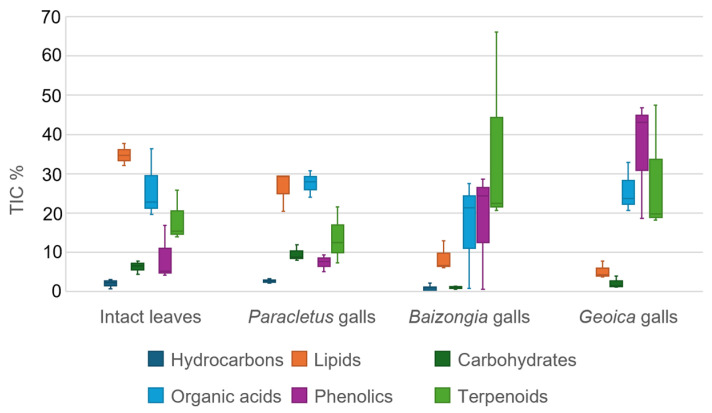
Distribution of total ion Current (TIC%) values (median and interquartile range (IQR)) for primary compound classes in EtOAc extracts.

The metabolic profiles of EtOAc extracts reveal a diverse range of compound classes, including hydrocarbons, lipids, carbohydrates, organic acids, phenolics, and terpenoids (Table S1, Figure 2). *Paracletus* galls closely resemble intact leaves but contain lower lipid and higher carbohydrate concentrations. In contrast, *Baizongia* and *Geoica* galls exhibit a distinct metabolic profile, characterized by reduced hydrocarbons, lipids, and carbohydrates, alongside elevated and more variable levels of phenolics and terpenoids.

**Figure 3:**
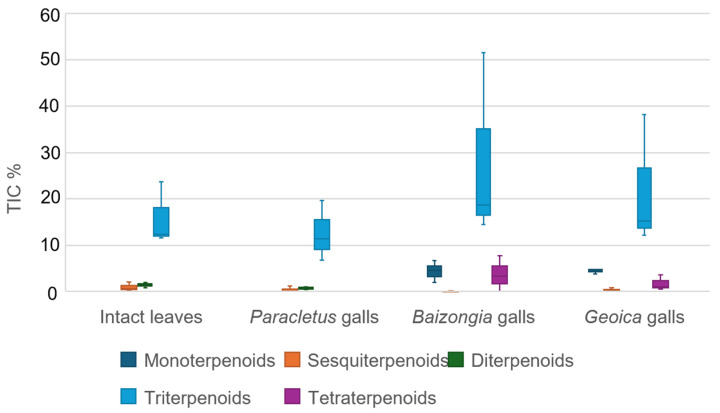
TIC% values (medians and IQR) for terpenoids in EtOAc extracts in galls and leaves.

Among the terpenoids, triterpenoids were the most abundant across all samples, with *Baizongia* galls showing the highest concentrations, followed by *Geoica* galls (Figure 3).

Monoterpenoids and the sole tetraterpenoid, neurosporaxanthin methyl ester, were detected exclusively in *Baizongia* and *Geoica* galls. Sesquiterpenoids were scarce and primarily found in intact leaves. The only identified diterpenoid, phytol—a chlorophyll degradation product—was more abundant in leaves but also present in *Paracletus* galls.

#### 2.1.1. Metabolic Variations in EtOAc Extracts Between Galls and Leaves

Principal component analysis (PCA) captured 100% of the variance in the median concentrations of individual metabolites, with the first three principal components ex-plaining the following proportions: F1 (70%), F2 (16%), and F3 (14%) (Figure S1, Table S3). Detailed factor loadings are provided in Table S4:

- F1: Metabolites with high positive loadings were predominant in intact leaves and *Paracletus* galls, while negative loadings were associated with *Baizongia* and *Geoica* galls.
- F2: This axis reflected the accumulation of α-terpinene, zonarene, and galactose/galactinol isomers in *Geoica* samples and gallic acid in *Paracletus*, *Baizongia*, and *Geoica* galls from Tree 1.
- F3: Negative loadings corresponded to higher fructose isomer levels in *Paracletus* galls and ursolic acid in *Baizongia* (Tree 2) and all *Geoica* samples.

**Figure 4:**
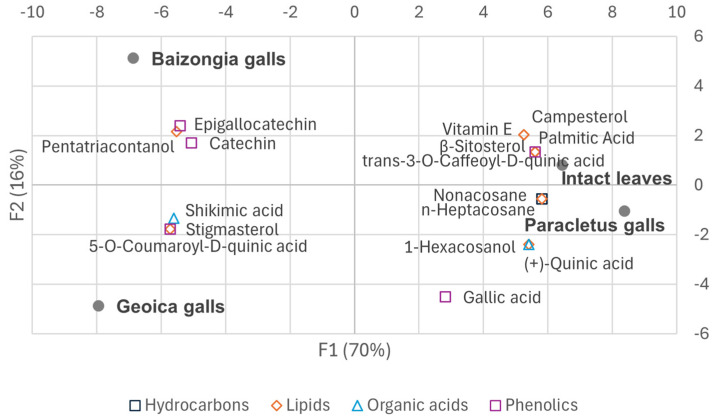
Biplot of samples and selected key metabolites from the primary compound classes from EtOAc extracts on principal component axes.

**Figure 5:**
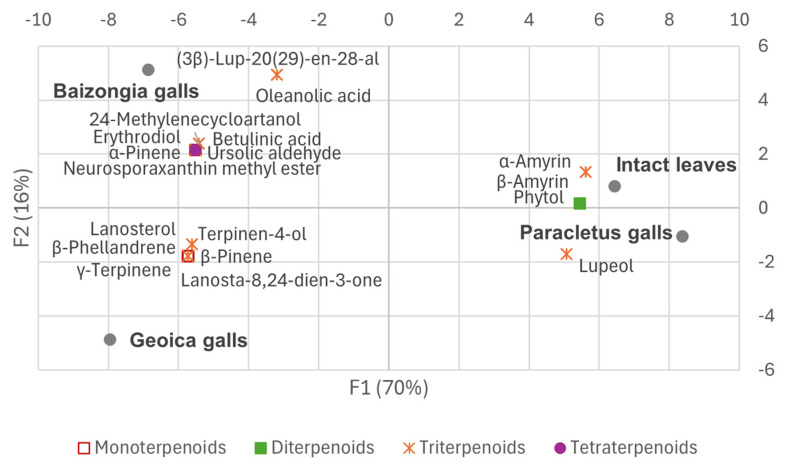
Biplot of samples and selected key metabolites from terpenoid sub-classes from EtOAc extracts on principal component axes.

The PCA biplots (Figure 4 and Figure 5) illustrate some of the key metabolites from the primary compound classes, emphasizing their role in sample differentiation.

**Figure 6:**
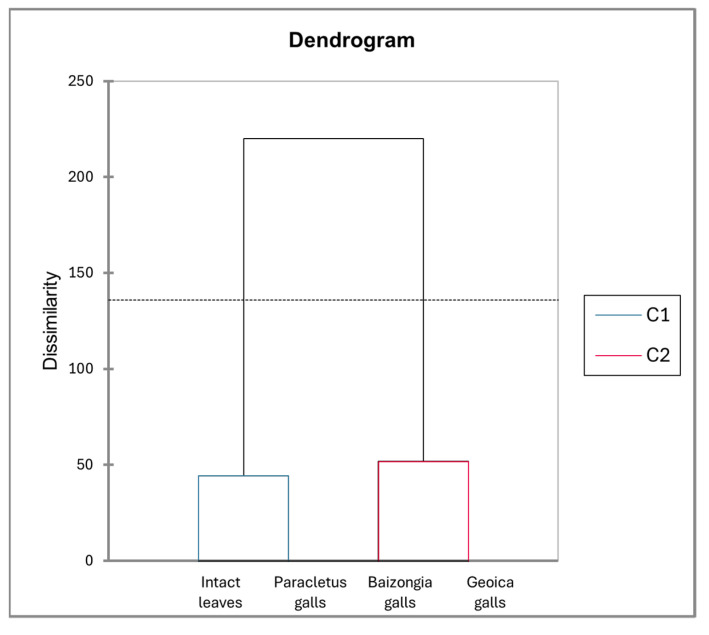
Hierarchical clustering of EtOAc extracts from leaves and galls based on PCA factor scores using Ward’s method.

The dendrogram derived from the PCA factor scores (Figure 6) reveals two distinct clusters: one comprising *Paracletus* galls and intact leaves and the other consisting of *Baizongia* and *Geoica* galls. This supports their metabolic similarity, as indicated in Figure 2 and the correlation analysis. The branch lengths emphasize the metabolic divergence among groups, underscoring the unique chemical profiles of each gall type and its aphid inducer.

#### 2.1.2. Tree-Specific Variations in the Metabolites of EtOAc Extracts from Galls and Leaves

The results of Kruskal–Wallis test, based on raw TIC% values of individual compounds, were not significant. However, Spearman correlation analysis across samples revealed a strong positive relationship between intact leaves and *Paracletus* galls (r_s_ = 0.830, *p* < 0.0001). On the other side, the metabolites found in *Baizongia* and *Geoica* galls were significantly correlated.

PCA further explored these associations, revealing distinct variations in standardized metabolite concentrations across trees. F1 and F2 together explained 61% of total variability (Figure S2, Table S5). Factor loadings (Table S6) indicated:

- F1: Terpenoids were enriched in *Baizongia* (T1B, T2B, T3B) and *Geoica* galls (T1G, T2G, T3G), whereas lipids were more abundant in *Paracletus* galls (T1P, T2P, T3P) and in-tact leaves (T1L, T2L, T3L). Additionally, moderate positive loadings were associated with terpenoids, including α-terpinene, terpinene-4-ol, lanosta-7,9,24-trien-3β-ol, and the sugar alcohol galactinol (isomer 2), which accumulated specifically in *Geoica* galls.
- F2: This axis lacked clear patterns, though negative loadings reflected certain com-pound accumulations in *Baizongia* and *Geoica* galls across trees. A strong positive loading corresponded to campesterol accumulation in *Paracletus* galls and leaves from all trees.

**Figure 7:**
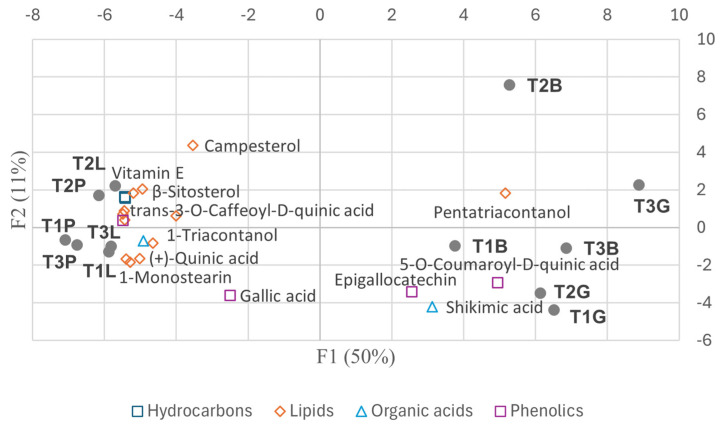
Biplot of selected individual metabolites from the classes of hydrocarbons, lipids, organic acids, and phenolic compounds, and their contribution to PCA variability among trees (EtOAc extracts). Abbreviations: T1–3 = individual trees; L = intact leaves; P = *Paracletus* galls; B = *Baizongia* galls; G = *Geoica* galls. Note that P and L exhibit greater similarity to each other, whereas B and G display distinct metabolic profiles.

**Figure 8:**
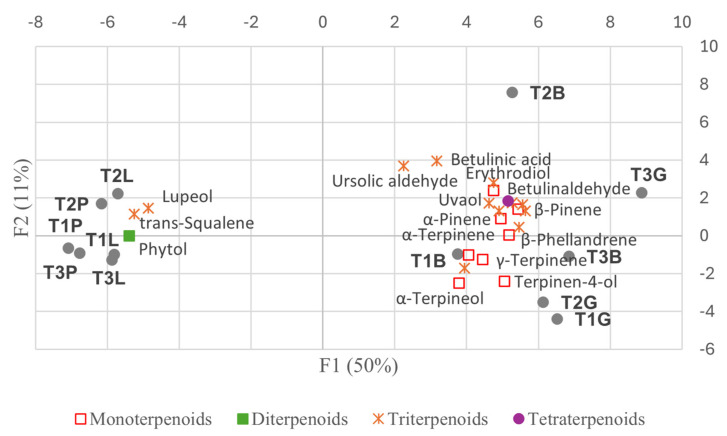
Biplot of selected individual terpenoids and their contribution to PCA variability among trees (EtOAc extracts). Abbreviations: T1–3 = individual trees; L = intact leaves; P = *Paracletus* galls; B = *Baizongia* galls; G = *Geoica* galls. Note that P and L exhibit greater similarity to each other, whereas B and G display distinct metabolic profiles.

The biplots (Figure 7 and Figure 8) highlight some key metabolites contributing to the observed variability.

**Figure 9:**
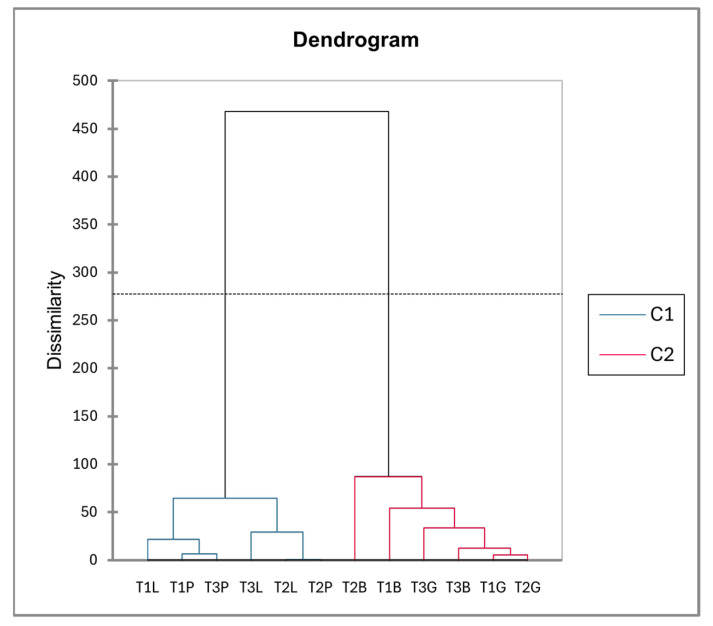
Hierarchical clustering of tree-specific EtOAc extracts based on PCA factor scores generated using Ward’s method. Abbreviations: T1–3 = individual trees; L = intact leaves; P = *Paracletus* galls; B = *Baizongia* galls; G = *Geoica* galls. Note that P and L exhibit greater similarity to each other, whereas B and G display distinct metabolic profiles.

The dendrogram (Figure 9) delineates clustering patterns among trees. The first cluster, containing intact leaves and *Paracletus* galls, reflects their close metabolic relationship, with some inter-tree variation. The second cluster groups *Baizongia* and *Geoica* galls.

### 2.2. Metabolic Profiles of MeOH Extracts from Galls and Leaves

**Figure 10:**
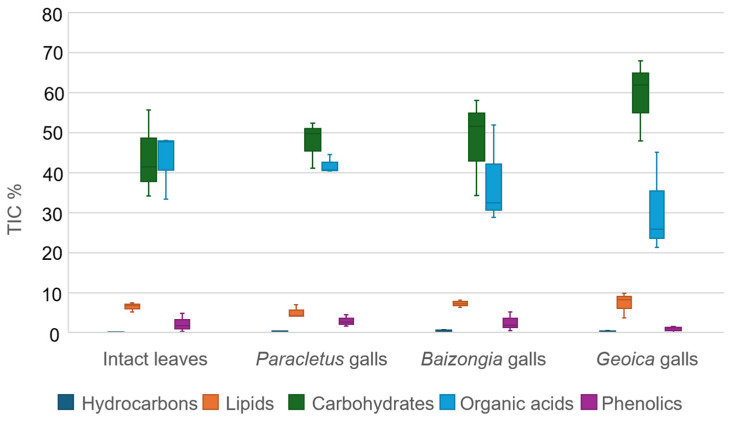
Distribution of TIC% values (median and IQR) for primary compound classes in MeOH extracts.

The main compound classes identified in the MeOH extracts following acid hydrolysis included hydrocarbons, lipids, carbohydrates, organic acids, and phenolics (likely re-leased as aglycones) (Table S2, Figure 10).

The metabolic profile of *Paracletus* galls closely resembled that of intact leaves, whereas *Baizongia* and particularly *Geoica* galls exhibited reduced levels of organic acids but increased carbohydrate content.

#### 2.2.1. Metabolic Variations in MeOH Extracts Between Galls and Leaves

PCA accounted for 100% of the variance in the median metabolite concentrations, with the first three components contributing as follows: F1 (51%), F2 (28%), and F3 (21%) (Figure S3, Table S7). Factor loadings (Table S8) indicated:

- F1: Metabolites with high positive loadings were mainly enriched in intact leaves and *Paracletus* galls, with some also present in *Baizongia* galls. Negative loadings corresponded to metabolites predominantly found in *Baizongia* and *Geoica* galls.
- F2: Positive loadings highlighted 2-monopalmitin and 1-monostearin in leaves, 2-keto-*L*-gluconic acid in *Baizongia* galls, and gluconic acid in *Paracletus* galls. Negative loadings corresponded to *n*-heptadecane, malic acid, pyrogallol, and coniferyl alcohol in *Paracletus* galls, and palmitic acid in *Geoica* galls.
- F3: Positive loadings indicated *n*-eicosane, galactose, methyl galactoside, sucrose, and caffeic acid in *Baizongia* galls, and fructose in *Geoica* galls. Negative loadings were linked to methyl glucoside, predominantly in leaves and *Paracletus* galls, and 4-*O*-methyl-myo-inositol in *Geoica* and *Paracletus* galls.

**Figure 11:**
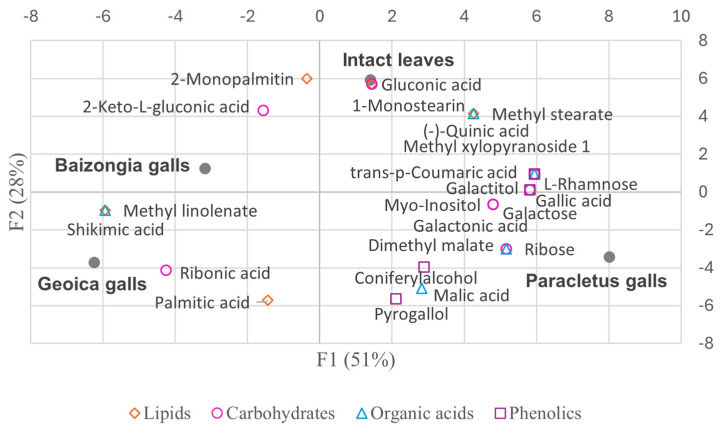
Biplot of samples and selected metabolites with meaningful variation from MeOH extracts on principal components.

Figure 11 presents a biplot showing the distribution of samples and selected metabolites with meaningful variation from the MeOH extracts, based on the first two principal components from the PCA.

**Figure 12:**
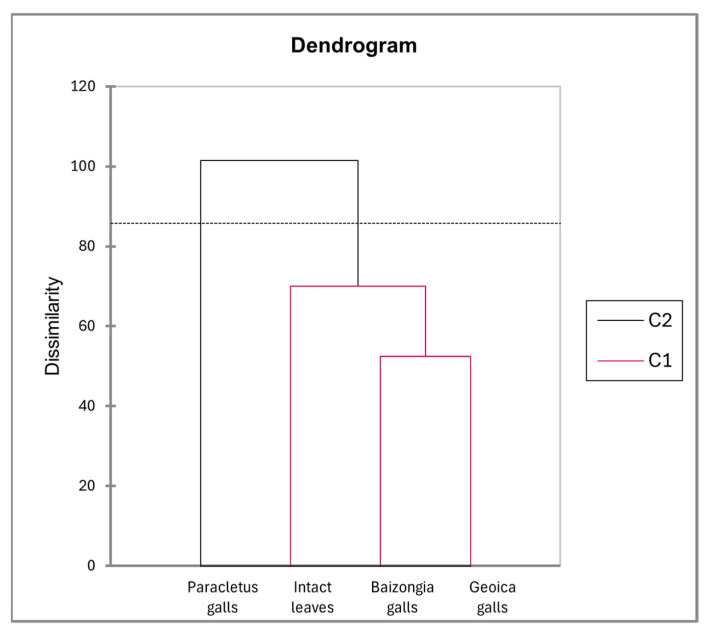
Hierarchical clustering of MeOH extracts from leaves and galls based on PCA factor scores using Ward’s method.

The dendrogram (Figure 12), derived from the PCA analysis, reveals two primary clusters (C2 and C1), reflecting distinct metabolic profiles among intact leaves and galls.

Cluster C2, consisting exclusively of *Paracletus* galls, indicates a unique metabolic profile. Cluster C1 includes intact leaves, *Baizongia* galls, and *Geoica* galls, with *Baizongia* and *Geoica* forming a well-defined sub-cluster, suggesting a strong metabolic similarity between them.

Although intact leaves are grouped within C1, their metabolic distance from *Baizongia* and *Geoica* galls appears similar to their distance from *Paracletus* galls in C2. This suggests that intact leaves share some degree of metabolic similarity with both groups, highlighting a gradient of metabolic divergence rather than a strict separation.

#### 2.2.2. Tree-Specific Variations in the Metabolites of MeOH Extracts from Galls and Leaves

The Kruskal–Wallis test (*p* << 0.05) confirmed significant metabolic differences among the samples, while the Bonferroni-corrected Dunn post hoc analysis (*p* << 0.01) revealed that these differences are driven by both aphid species and tree-specific factors, resulting in distinct metabolic profiles among gall types (Table 1).

Aphid species strongly influence metabolic profiles (Table 1). *Paracletus* galls (T1P, T2P, T3P) consistently cluster closer to intact leaves (T1L, T2L, T3L), as indicated by their similar rank values and shared statistical groups, suggesting minimal metabolic divergence and a chemical composition that largely resembles ungalled tissue. In contrast, *Geoica* (T1G, T2G, T3G) and *Baizongia* galls (T1B, T2B, T3B) exhibit more pronounced metabolic shifts, forming distinct clusters that separate from both intact leaves and *Paracletus* galls.

Tree-specific effects further modulate these aphid-induced changes (Table 1). Among *Geoica* galls, T2G (Tree 2) exhibits the lowest rank value, indicating the strongest metabolic divergence in this tree. Similarly, T3B (*Baizongia* gall in Tree 3) undergoes the most pronounced metabolic shifts, differing significantly from multiple other samples, suggesting a greater metabolic response in this tree. In contrast, *Baizongia* and *Geoica* galls from Trees 1 and 2 exhibit fewer significant differences, indicating a less pronounced metabolic shift in these trees.

The relatively consistent metabolic profile of intact leaves across trees (T1L, T2L, T3L) reinforces their chemical stability, while the minimal metabolic impact of *Paracletus* galls suggests that their formation preserves a metabolic environment similar to ungalled tis-sue, regardless of tree origin. The clustering pattern (Table 1) highlights a gradient of metabolic divergence, with *Paracletus* galls remaining the most similar to intact leaves, while *Geoica* and *Baizongia* galls undergo stronger metabolic reprogramming.

PCA of the standardized raw concentrations of individual compounds revealed dis-tinct metabolic variations among trees, with F1, F2, and F3 collectively explaining 63% of the total variability (Figure S4, Table S9). The corresponding loadings (Table S10) highlight key metabolites contributing to these differences:

- F1 captures a complex distribution of metabolites, with some enriched in a single tree, while others are shared between two. Negative loadings include shikimic acid and methyl linolenate, both enriched in Tree 3, and melibiose, which is more abundant in Tree 1.
- F2 primarily reflects metabolites associated with Tree 1, with the only negative loading corresponding to methyl glucoside, which is enriched in Tree 2.
- F3 differentiates Tree 2 and Tree 3, where positive loadings correspond to metabolites enriched in Tree 2, while negative loadings represent compounds predominantly found in Tree 3.

**Figure 13:**
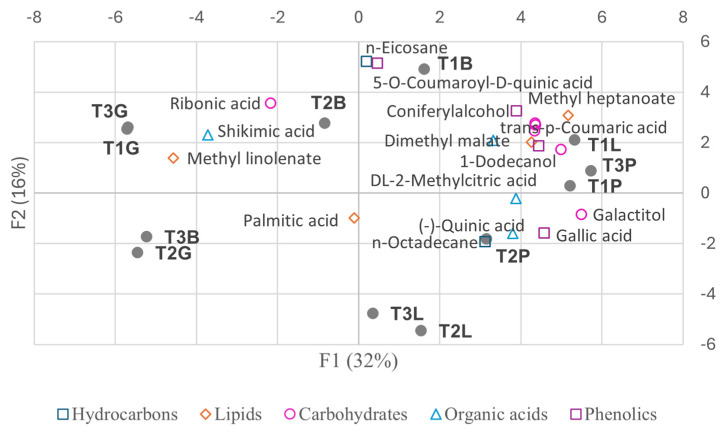
Biplot of selected individual metabolites and their contribution to PCA variability among trees (MeOH extracts). See abbreviations in Figure 7.

The biplot (Figure 13) further highlights key compounds with high loadings that con-tribute to this metabolic variability, reinforcing the observed aphid- and tree-specific metabolic patterns.

**Figure 14:**
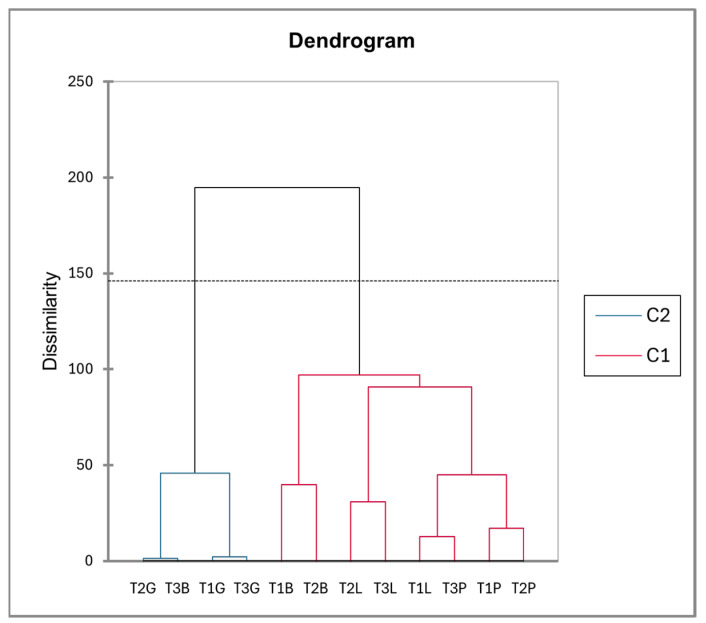
Hierarchical clustering of tree-specific MeOH extracts based on PCA factor scores using Ward’s method. See abbreviations in Figure 7.

The dendrogram based on PCA factor scores (Figure 14) supports both aphid- and tree-specific patterns.

*Geoica* galls form a cohesive cluster, indicating a largely conserved metabolic profile across trees. However, within this cluster, samples from Trees 1 (T1G) and 3 (T3G) are more similar, while those from Tree 2 (T2G) diverge slightly, suggesting subtle tree-specific metabolic differences despite the overall aphid-driven signature.

A similar pattern is observed in intact leaves, where those from Trees 2 and 3 cluster together, while Tree 1 remains distinct. This suggests that Trees 2 and 3 share metabolic characteristics, which also influence *Geoica* galls, reinforcing the role of host tree factors in shaping gall metabolism.

In contrast, galls induced by *Paracletus* and *Baizongia* exhibit stronger tree-specific effects. Those from Tree 1 cluster closely with those from Tree 2, whereas Tree 3 is distinctly separated, indicating that this host experiences the most pronounced metabolic shifts in these aphid-induced structures. This pattern aligns with PCA results, which highlight Tree 3 as metabolically distinct, particularly in *Baizongia* (T3B). However, Dunn post hoc analysis shows that *Geoica* galls from Tree 2 (T2G) have the highest number of significant differences, suggesting that while T3B is highly divergent within *Baizongia*, T2G represents the strongest metabolic shift overall.

Overall, the dendrogram highlights a gradient of metabolic variation, where *Geoica* galls maintains a more conserved profile but still exhibits tree-specific differences, particularly in Tree 2. Meanwhile, *Baizongia* and *Paracletus* galls show stronger host-driven metabolic divergence, with Tree 3 displaying the most distinct shifts.

## 3. Discussion

### 3.1. Limited Host Impact and Subtle Manipulation by P. cimiciformis

Our study reveals that *P. cimiciformis* induces galls with relatively minor anatomical and metabolic disruption to its host. Despite their unique chemical composition, these galls’ metabolic profile closely resembles that of intact leaves, reflecting subtle integration into the host plant’s structure with limited interference in natural processes (Figure 6 and Figure 12). This weak manipulation, however, limits the number of aphids that can develop within each simple gall, typically to just a few dozen.

Metabolic adjustments in *Paracletus* galls are finely tuned (Tables S1 and S2, Figure 4, Figure 5 and Figure 11). Increased levels of cuticular wax components such as *n*-heptadecane, *n*-eicosane, and nonacosane enhance structural integrity, providing protection against desiccation, UV radiation, and microbial threats [18]. Elevated levels of α-amyrin and lupeol, triterpenoids associated with plant defense [19,20], further strengthen the gall’s resistance to environmental stress.

An unusual accumulation of free myristic acid—rarely found in high concentrations in plants [21]—is observed in *Paracletus* galls of Trees 1 and 3 (Table S1). This points to alterations in lipid metabolism or membrane structure, potentially boosting the gall’s resilience and supporting aphid nutrition. *N*-terminal myristoylation of plant proteins, crucial for membrane targeting and signal transduction in stress responses, may be a key factor in these changes [22].

Despite reductions in key antioxidants such as vitamin E and shikimic acid, the galls exhibit increased levels of (+)-quinic and gallic acids (Tables S1 and S2), both of which are known for their antioxidant and antimicrobial properties [23]. These shifts help balance the need to maintain a metabolic profile similar to that of intact leaves with fulfilling the aphid’s nutritional and ecological requirements.

Elevated sugars may further support aphid feedingand survival. Ribose, essential for nucleotide biosynthesis and energy metabolism, sustains cellular processes [24]. Ribonic acid, along with the osmolytes myo-inositol and melibiose, aids stress adaptation under varying conditions [25,26,27].

These subtle metabolic shifts enable *P. cimiciformis* to create a microenvironment that supports its performance while preserving the characteristics of intact leaf tissue. However, the limited alterations to the host plant constrain the galls’ capacity to sustain many aphids. 

### 3.2. Manipulation of Host Terpenoid Metabolism by B. pistaciae and Geoica spp.

*Baizongia* and *Geoica* aphids manipulate host plant terpenoid metabolism to fortify gall structures, which are larger and more complex than those induced by *P. cimiciformis*. These aphid-induced galls are enriched in triterpenoids, including lanostane, cycloartane, ursane, and oleanane types (Table S1). Synthesized in the cytoplasm or endoplasmic reticulum and stored in vacuoles or glandular trichomes, these compounds create a robust protective barrier on the gall surface, shielding against natural enemies and environmental stressors [28,29].

Interestingly, neurosporaxanthin methyl ester, a tetraterpenoid pigment typically as-sociated with fungi such as *Fusarium* and *Neurospora*, was also detected in *Baizongia* and *Geoica* galls. *Pistacia* is one of the few plant species capable of producing this compound. Its presence in the galls suggests roles in pigmentation, UV protection, and enhanced chemical defense [30,31].

### 3.3. Shikimate and Phenylpropanoid Pathway Shifts Induced by Baizongia and Geoica Aphids

Terpenoids and phenolic compounds synergistically enhance gall resilience [32]. Phenolics regulate microbial activity in the carbohydrate-rich environment created by aphid sap feeding [33], interact with reactive oxygen species, and modulate auxins like indole-3-acetic acid, influencing gall growth and development [34,35]. This interaction, possibly involving auxin-(poly)phenol adducts, positions phenolics primarily as growth regulators and secondarily as chemical defenses against natural enemies, heat, and UV radiation, thereby stabilizing galls metabolically and defensively [35,36].

The activation of the shikimate and phenylpropanoid pathways in *Baizongia* and *Geoica* galls further strengthens this defense system by accumulating phenolic antioxidants such as gallic acid, catechin, epigallocatechin, and 5-*O*-*p*-coumaroyl-D-quinic acid (Tables S1 and S2; Figure 4, Figure 5 and Figure 11) [33,35,37,38]. 

Interestingly, the accumulation of 5-*O*-*p*-coumaroyl-D-quinic acid in *Baizongia* and *Geoica* galls, alongside the suppression of *trans*-3-*O*-caffeoyl-D-quinic acid (chlorogenic acid), suggests a specific metabolic reprogramming that redirects phenylpropanoid flux toward *p*-coumaroyl derivatives. This shift may serve to reinforce gall structural integrity while modulating plant defense responses.

### 3.4. Metabolic Variation Across Aphid Species and Host Trees

Our analysis uncovered distinct metabolic profiles in galls shaped by both aphid-induced changes and tree-specific factors. In the EtOAc extracts, *Paracletus* galls exhibited a metabolic profile more akin to that of intact leaves, though distinct variations were observed between tree samples, emphasizing the role of tree identity in shaping metabolite composition (Figure 8, Table S7).

The metabolic profiles of *Baizongia* galls were similar to those of *Geoica* galls, suggesting that these two aphid species induce comparable metabolic shifts in the host plant (Figure 9). However, differences between trees also played a role, as *Geoica* galls from Trees 1 and 2 were more similar to each other than to those from Tree 3, indicating subtle host-specific metabolic variations. A similar pattern of inter-tree differences was observed in *Baizongia* galls, as reported in other studies [8,10,12].

In MeOH extracts, *Paracletus* galls remained metabolically closest to intact leaves and, along with *Baizongia* galls, exhibited a stronger host-driven metabolic divergence. In contrast, *Geoica* galls maintained consistent metabolic profiles across all trees, suggesting a reduced sensitivity to host tree variations (Figure 14). Although metabolite accumulation varied among trees, most metabolites were associated with a distinct gall type (Table S10, Figure 13).

### 3.5. Interplay Between Aphid Species, Host Genotype, and Environment

Our findings highlight the interplay between aphid species and host plant genotypes, where gall phenotype is shaped not only by insect-induced changes but also by the genetic framework of the host plant. The distinct metabolic profiles observed across different trees point to intricate cross-talk between aphids and plant genotypes.

Undoubtedly, environmental factors, phenological phases, and intraspecific genetic and phenotypic diversity of both aphids and plants influence the composition and quantity of gall metabolites [8,39]. Gall-forming insects hijack the genetic and metabolic pathways of their host plants [40], with more structurally and anatomically complex galls exhibiting greater metabolic divergence from intact leaves. In the future, it will be interesting to link our findings with the detailed anatomical structure of gall induced by different aphid species (see [41]).

Galls induced by Fordini aphids are particularly rich in bioactive compounds, which strengthen structural integrity and enhance defense mechanisms. This discovery suggests potential applications of these unique plant formations in biocides, eco-friendly pesticides, and natural additives for the pharmaceutical, cosmetic, and food preservation industries [42].

### 3.6. Comparison of EtOAc and MeOH Extracts

In addition to aphid- and tree-driven metabolic variations, differences between solvent extraction methods also provided important insights into the composition of gall and leaf metabolites. A comparative analysis of EtOAc and MeOH extracts revealed key trends in compound class distribution. EtOAc extracts were particularly enriched in terpenoids and other lipophilic compounds, whereas MeOH extracts contained higher proportions of polar metabolites, including phenolics, carbohydrates, and organic acids.

Despite these solvent-specific differences, both extraction methods highlighted metabolic shifts between galls and leaves. Certain compounds, including shikimic acid and quinic acid, which were among the most abundant metabolites, were consistently detected in both solvents, though their levels varied between extracts. In contrast, terpenoids were exclusively identified in EtOAc extracts, underscoring their strong association with lipophilic fractionation.

This comparative approach enhances our understanding of gall metabolism by demonstrating how different solvent extraction methods capture complementary aspects of plant biochemistry. The observed differences emphasize the role of solvent polarity in detecting distinct compound classes, reinforcing the complex metabolic landscape shaped by both aphid species and host tree genotypes.

## 4. Materials and Methods

### 4.1. Plant Material

Plant material was collected on 14 August 2023, from three wild *P. palaestina* trees located in Shchania (Tree 1), Beit Govrin (Tree 2), and Aninadav (Tree 3), Israel. The samples comprised intact leaves (T1L, T2L, and T3L) and galls induced by *P. cimiciformis* (T1P, T2P, and T3P), *B. pistaciae* (T1B, T2B, and T3B), and *Geoica* spp. (T1G, T2G, and T3G). All samples were stored at −20 °C until analysis.

### 4.2. Extraction

Frozen samples were ground to fine powder in liquid nitrogen. To remove any insect remains, the galls were cut in half and brushed clean. One hundred milligrams of the prepared plant material were sequentially extracted with 1 mL of EtOAc followed by 1 mL of MeOH, each for 24 h. Solvents were evaporated under a stream of nitrogen gas. Additionally, MeOH extracts underwent hydrolysis in a concentrated HCl/MeOH solution at 60 °C.

### 4.3. Sample Preparation for GC–MS Analysis

For GC–MS analysis, 5 mg of each EtOAc and hydrolyzed MeOH extract was silylated by dissolving the sample in 100 µL of pyridine, followed by the addition of 100 µL of *N*,*O*-bis(trimethylsilyl) trifluoroacetamide. The mixture was incubated at 80 °C for 1 h. After silylation, the EtOAc extracts (25 µL) were diluted in 275 µL of chloroform, while the MeOH extracts (50 µL) were dissolved in 250 µL of chloroform.

### 4.4. GC–MS Analysis

GC-MS analysis was conducted using an Agilent 7890A (Agilent Technologies Inc., Santa Clara, CA, USA) gas chromatograph coupled with an Agilent 5975C (Agilent Technologies Inc., Santa Clara, CA, USA) mass selective detector. The system employed a DB-5ms silica-fused capillary column with a stationary phase of poly(dimethylsiloxane) (30 m × 0.25 mm i.d., 0.25 µm film thickness). The oven temperature program was set as follows: the initial temperature was maintained at 40 °C for 5 min, followed by a ramp of 5 °C/min to 300 °C, where it was held for an additional 15 min, resulting in a total run time of 70 min. Helium was used as the carrier gas at a flow rate of 0.8 mL/min. The injector and transfer line temperatures were set at 250 °C, and the MS source temperature was maintained at 230 °C. Injections were performed in splitless mode with a volume of 1 µL.

Relative retention indices (RIs) were calculated using a standard mixture of aliphatic hydrocarbons (C10–C40, Sigma, Setagaya City, Japan), injected under the same temperature program. Metabolites were identified by comparing retention times and RIs with those of authentic standards, and by matching spectral data with the National Institute of Standards and Technology (NIST 08) libraries [43], the Golm Metabolome Database (GMD) [44], and other literature sources [45].

### 4.5. Data Analysis

Data analysis was conducted using XLSTAT 2024.2.2, focusing on the TIC% of individual compounds in the extracts (Tables S1 and S2) to evaluate chemical composition distribution across the samples.

The TIC% presented in Figure 2, Figure 3 and Figure 10 was calculated after removing outliers, as described in Section 4.5.1. The remaining compounds were classified into structural categories using spectral library matching (NIST/Wiley, Hoboken, NJ, USA), literature references, and expert evaluation.

Figure 2: Compounds were grouped into hydrocarbons, lipids, carbohydrates, organic acids, phenolics, and terpenoids.

Figure 3: Terpenoids were further categorized into mono-, sesqui-, di-, tri-, and tetraterpenoids.

Figure 10: Compounds were grouped into hydrocarbons, lipids, carbohydrates, organic acids, and phenolics.

For all figures, the TIC% for each structural class was determined by summing the raw TIC% of all individual compounds within that class.

#### 4.5.1. Descriptive Statistics and Outlier Detection

Descriptive statistics, including median and interquartile range (IQR), were calculated to assess the central tendency and variability of chemical compositions. Box plots were generated to visualize and identify outliers, which were subsequently removed.

#### 4.5.2. Normality Assessment

The normality of raw data was assessed using the Shapiro–Wilk test and visual inspection of Q–Q plots. Deviations from normality necessitated the use of non-parametric statistical tests.

#### 4.5.3. Kruskal–Wallis Test

The Kruskal–Wallis test was applied to the raw TIC% values of individual metabolites to assess differences among sample groups. When significant differences were detected (*p* < 0.05), Dunn’s post hoc test with Bonferroni correction was performed for pairwise comparisons to account for multiple testing.

#### 4.5.4. Correlation Test

Spearman correlation analysis was performed on the TIC% values of individual compounds to evaluate metabolic relationships across samples. Analyses were conducted separately for EtOAc and MeOH extracts to assess correlation patterns. High multicollinearity in MeOH extracts led to the application of principal component analysis (PCA) as a dimensionality reduction method to improve data interpretability.

#### 4.5.5. Data Standardization

Following outlier removal and distribution assessment, raw values were standardized into z-scores to facilitate meaningful comparisons. This transformation minimized bias from differences in value magnitudes, enhancing comparability across compounds.

#### 4.5.6. PCA

PCA was employed to reduce dataset dimensionality while preserving variance. This transformation converted correlated variables into uncorrelated components, addressing multicollinearity. PCA aimed to reveal core structural patterns in the data, enhancing interpretability and facilitating sample differentiation.

For PCA analysis, different preprocessing approaches were applied depending on the comparison type. When analyzing differences between leaves and gall types, TIC% values for each compound were first aggregated by calculating the median TIC% within each leaf and gall group. This was followed by normalization and PCA, ensuring that group-level metabolic trends were maintained while minimizing the influence of extreme values.

In contrast, when comparing metabolic variation between trees, raw TIC% values were used, with normalization applied only before PCA analysis. This approach preserves tree-specific metabolic variability while avoiding potential biases from prior aggregation. These distinct preprocessing strategies were chosen to enhance the biological interpretability of the results.

#### 4.5.7. AHC

To complement PCA, AHC was employed to classify samples based on compound profiles. The Ward method was used to minimize within-cluster variance, and Euclidean distances were applied to measure dissimilarity. This hierarchical clustering approach iteratively merged similar clusters, uncovering natural groupings. The resulting dendrograms provided insights into sample relationships and facilitated cluster identification based on compound composition.

## 5. Conclusions

This study provides new insights into the metabolic modifications induced by gall-forming aphids on *P. palaestina*. By integrating GC–MS metabolite profiling with comparative analyses, we revealed that gall formation induces species-specific and tree-dependent metabolic shifts, reshaping both lipophilic and hydrophilic metabolite pools.

*Paracletus* galls exhibited a metabolic profile more similar to that of intact leaves, suggesting a subtle form of host manipulation, whereas *Baizongia* and *Geoica* galls triggered more pronounced metabolic alterations, particularly in terpenoid and phenolic pathways. Tree identity further influenced gall metabolite composition, underscoring the role of host genotype in shaping gall biochemistry.

Additionally, solvent-based extraction revealed complementary aspects of gall metabolism. EtOAc extracts were enriched in terpenoids and other lipophilic compounds, whereas MeOH extracts contained higher proportions of polar metabolites such as phenolics and carbohydrates. Despite these solvent-dependent differences, several key metabolites—including shikimic acid and quinic acid—were consistently detected in both extracts, highlighting core metabolic adaptations associated with gall formation.

These findings contribute to our understanding of plant–insect interactions, demonstrating how aphids modulate host metabolism to establish a specialized microenvironment within galls. The observed species-specific and genotype-dependent metabolic shifts may indicate co-evolutionary processes at the metabolic level between *P. palaestina* and its gall-forming aphids. However, confirming this hypothesis will require further genetic and ecological studies to elucidate the evolutionary and adaptive significance of these biochemical interactions.

Moreover, the discovery of bioactive compounds in these galls suggests potential applications in biocides, pharmaceuticals, and food preservation industries. Further research is needed to explore the functional roles of these metabolites and their potential ecological and biotechnological significance.

## Figures and Tables

**Table 1:** Pairwise multiple comparisons of metabolite profiles of MeOH extracts using Dunn’s two-tailed test. Mean rank values indicate metabolic differences among samples. Letters (A, B, C) represent statistically distinct groups, where samples sharing the same letter are not significantly different. Lower rank values indicate greater metabolic divergence.

Sample	Mean of Ranks	Groups		
T2G	252.212	A		
T3B	266.144	A	B	
T3G	295.288	A	B	C
T3L	322.763	A	B	C
T1G	326.322	A	B	C
T2L	365.864	A	B	C
T2P	386.814		B	C
T2B	393.254			C
T1B	401.424			C
T1P	412.407			C
T1L	415.186			C
T3P	416.322			C

## Data Availability

The original contributions presented in this study are included in the article/Supplementary Materials. Further inquiries can be directed to the corresponding authors.

## Supplementary Materials

The following supporting information can be downloaded at: https://www.mdpi.com/article/10.3390/plants14050721/s1, Figure S1: Scree plot of principal components showing explained variance for compounds in EtOAc extracts across samples of leaves and galls induced by *Paracletus*, *Baizongia*, and *Geoica* aphids; Figure S2: Scree plot of principal components and explained variance for compounds in EtOAc extracts of leaves and galls induced by *Paracletus*, *Baizongia*, and *Geoica* aphids across trees; Figure S3: Scree plot of principal components showing explained variance for compounds in MeOH extracts across samples of leaves and galls induced by *Paracletus*, *Baizongia*, and *Geoica* aphids; Figure S4: Scree plot of principal components and explained variance for compounds in MeOH extracts across samples of leaves and galls induced by *Paracletus*, *Baizongia*, and *Geoica* aphids across trees; Table S1: GC–MS profiling of compounds in EtOAc extracts of *Pistacia palaestina* leaves and galls induced by *Paracletus* (P), *Baizongia* (B), and *Geoica* (G) across three trees (T1, T2, and T3); Table S2: GC–MS profiling of compounds in MeOH extracts of *Pistacia palaestina* leaves and galls induced by *Paracletus* (P), *Baizongia* (B), and *Geoica* (G) across three trees (T1, T2, and T3); Table S3: Principal components summary, eigenvalues, and variance explained for compounds in EtOAc extracts across samples; Table S4: PCA factor loadings highlighting variation in median TIC% of individual compounds between leaves and galls induced by *Paracletus*, *Baizongia*, and *Geoica* aphids (EtOAc extracts) (values in bold correspond to the factor for which the squared cosine is the largest); Table S5: Summary of principal components, eigenvalues, and variance contributions for compounds across trees (EtOAc extracts); Table S6: PCA factor loadings highlighting variation in raw TIC% of individual compounds in EtOAc extracts of leaves and galls induced by *Paracletus*, *Baizongia*, and *Geoica* aphids across trees (values in bold correspond to the factor for which the squared cosine is the largest); Table S7: Summary of principal components, eigenvalues, and variance explained for compounds across samples (MeOH extracts); Table S8: PCA factor loadings highlighting variation in median TIC% of individual compounds between leaves and galls of *Paracletus*, *Baizongia*, and *Geoica* (MeOH extracts) (values in bold correspond to the factor for which the squared cosine is the largest); Table S9: Summary of principal components, eigenvalues, and variance contributions for compounds across trees (MeOH extracts); Table S10: PCA factor loadings highlighting variation in raw TIC% of individual compounds in MeOH extracts of leaves and galls induced by *Paracletus*, *Baizongia*, and *Geoica* aphids across individual trees (T1, T2, and T3) (values in bold correspond to the factor for which the squared cosine is the largest).
