# Supplementary material for: Association Between Gall Structural and Metabolic Complexity: Evidence from Pistacia palaestina
Source: Plants (Basel). 2025 Feb 26;14(5):721. doi: 10.3390/plants14050721 (PMC11901957; doi:10.3390/plants14050721)
Supplement: Supplementary file 1 [file plants-14-00721-s001.zip › plants-3488292-supplementary.pdf]

**Table S1.** GC/MS profiling of compounds in EtOAc extracts of *Pistacia palaestina* leaves and galls induced by *Paracletus* (P), *Baizongia* (B), and *Geoica* (G) across three trees (T1, T2, and T3).

| RT* | Name | RIcalc** | RIlit*** | T1L | T2L | T3L | T1P | T2P | T3P | T1B | T2B | T3B | T1G | T2G | T3G |
|---|---|---|---|---|---|---|---|---|---|---|---|---|---|---|---|
| 8.56 | Butyric acid, 1TMS | 904 | 890 | 0**** | 0 | 0 | 0 | 0 | 0 | 0.46 | 0 | 0 | 0 | 0 | 0 |
| 9.52 | α-Thujene | 935 | 939 | 0 | 0 | 0 | 0 | 0 | 0 | 1.73 | 0 | 0 | 0 | 0 | 0 |
| 9.73 | α-Pinene | 941 | 939 | 0 | 0 | 0 | 0 | 0 | 0 | 0 | 5.02 | 1.13 | 0.82 | 1.68 | 0.65 |
| 10.12 | Camphene | 950 | 946 | 0 | 0 | 0 | 0 | 0 | 0 | 0.45 | 0 | 0 | 0 | 0 | 0.10 |
| 10.79 | Sabinene | 973 | 969 | 0 | 0 | 0 | 0 | 0 | 0 | 0 | 0.12 | 0 | 0 | 0 | 0.60 |
| 10.90 | β-Pinene | 977 | 972 | 0 | 0 | 0 | 0 | 0 | 0 | 0.44 | 1.20 | 0.29 | 0.24 | 0.91 | 0.74 |
| 11.46 | β-Myrcene | 992 | 988 | 0 | 0 | 0 | 0 | 0 | 0 | 0 | 0 | 1.04 | 0 | 0 | 0.48 |
| 12.18 | α-Terpinene | 1018 | 1014 | 0 | 0 | 0 | 0 | 0 | 0 | 0 | 0 | 0.09 | 0.20 | 0 | 0.22 |
| 12.60 | β-Phellandrene | 1026 | 1022 | 0 | 0 | 0 | 0 | 0 | 0 | 0 | 0.19 | 0.93 | 0.16 | 0.47 | 0.43 |
| 12.70 | Limonene | 1030 | 1025 | 0 | 0 | 0 | 0 | 0 | 0 | 0.17 | 0 | 0 | 0 | 0 | 0 |
| 13.51 | γ-Terpinene | 1058 | 1054 | 0 | 0 | 0 | 0 | 0 | 0 | 0.35 | 0 | 0.59 | 0.69 | 0 | 0.72 |
| 13.59 | Lactic acid, 2TMS | 1063 | 1066 | 0 | 0.73 | 0.35 | 0.86 | 0.45 | 0 | 0 | 0.24 | 0 | 0 | 0.16 | 0 |
| 14.34 | Terpinolene | 1090 | 1086 | 0 | 0 | 0 | 0 | 0 | 0 | 0 | 0 | 0 | 0.08 | 0 | 0 |
| 17.29 | Terpinen-4-ol | 1178 | 1174 | 0 | 0 | 0 | 0 | 0 | 0 | 1.06 | 0 | 1.43 | 2.49 | 0.30 | 1.60 |
| 17.70 | α-Terpineol | 1191 | 1186 | 0 | 0 | 0 | 0 | 0 | 0 | 0 | 0 | 0 | 0.11 | 0.48 | 0.10 |
| 18.44 | *exo*-Borneol, 1TMS | 1224 | 1224 | 0 | 0 | 0 | 0 | 0 | 0 | 0 | 0 | 0.09 | 0 | 0.25 | 0 |
| 19.33 | *cis*-*p*-Menth-2-en-1-ol | 1256 | 1260 | 0 | 0 | 0 | 0 | 0 | 0 | 0 | 0 | 0 | 0 | 0 | 0.09 |
| 19.58 | Caprylic acid, 1TMS | 1262 | 1265 | 0 | 0 | 0 | 0.56 | 0 | 0 | 0 | 0 | 0 | 0.09 | 0 | 0 |
| 19.79 | *trans*-*p*-Menth-2-en-1-ol | 1270 | 1268 | 0 | 0 | 0 | 0 | 0 | 0 | 0 | 0 | 0 | 0 | 0 | 0.08 |
| 19.89 | Glycerol, 3TMS | 1274 | 1277 | 0 | 0.45 | 0.52 | 0.73 | 0.23 | 0.57 | 0.25 | 0.10 | 0.09 | 0.14 | 0 | 0.10 |
| 20.00 | *trans*-Sabinyl acetate | 1283 | 1285 | 0 | 0 | 0 | 0 | 0 | 0 | 0.10 | 0 | 0 | 0 | 0 | 0 |
| 20.17 | Bornyl acetate | 1286 | 1287 | 0 | 0 | 0 | 0 | 0 | 0 | 0.14 | 0.32 | 0.08 | 0.13 | 0 | 0.29 |
| 20.42 | (-)-Myrtenol, 1TMS | 1298 | 1302 | 0 | 0 | 0 | 0 | 0 | 0 | 0.09 | 0 | 0 | 0 | 0 | 0 |
| 21.86 | α-Cubebene | 1347 | 1345 | 0 | 0 | 0 | 0 | 0 | 0 | 0 | 0 | 0.13 | 0 | 0 | 0.47 |
| 22.46 | *trans*-Erythrono-1,4-lactone, 2TMS | 1381 | 1389 | 0 | 0.34 | 0.24 | 0 | 0 | 0 | 0 | 0 | 0 | 0 | 0 | 0 |
| 23.78 | β-Caryophyllene | 1422 | 1417 | 0.29 | 0 | 0.84 | 0 | 0.34 | 0 | 0 | 0 | 0 | 0 | 0 | 0.21 |
| 24.52 | Humulene | 1455 | 1452 | 0 | 0 | 0.34 | 0 | 0 | 0 | 0 | 0 | 0 | 0 | 0 | 0.11 |

| | | | | | | | | | | | | | | | |
|---|---|---|---|---|---|---|---|---|---|---|---|---|---|---|---|
| 24.69 | *trans*-Cadina-1(6),4-diene | 1470 | 1475 | 0 | 0 | 0 | 0 | 0 | 0 | 0 | 0 | 0 | 0 | 0 | 0.18 |
| 25.33 | Germacrene D | 1481 | 1484 | 0 | 0.29 | 0.66 | 0 | 0.62 | 0 | 0 | 0 | 0 | 0 | 0 | 0.10 |
| 25.47 | Malic acid, 3TMS | 1495 | 1497 | 0 | 0 | 0 | 0.28 | 0 | 0 | 0.49 | 0 | 0 | 0 | 0 | 0 |
| 25.60 | *trans*-Muurola-4(14),5-diene | 1498 | 1500 | 0 | 0 | 0 | 0 | 0 | 0 | 0 | 0 | 0 | 0 | 0 | 0.26 |
| 25.70 | α-Muurolene | 1503 | 1507 | 0 | 0 | 0 | 0 | 0 | 0 | 0 | 0 | 0 | 0 | 0 | 0.12 |
| 26.22 | δ-Cadinene | 1518 | 1522 | 0 | 0 | 0 | 0 | 0 | 0 | 0 | 0 | 0 | 0 | 0.11 | 0.22 |
| 26.33 | Zonarene | 1526 | 1528 | 0 | 0 | 0 | 0 | 0 | 0 | 0 | 0 | 0.12 | 0 | 0.13 | 0.34 |
| 26.56 | Pyrogallol, 3TMS | 1533 | 1537 | 0 | 0.37 | 0.48 | 0.26 | 0 | 0 | 0 | 0 | 0.10 | 0 | 0.08 | 0.29 |
| 26.61 | α-Calacorene | 1543 | 1544 | 0 | 0 | 0 | 0 | 0 | 0 | 0 | 0.15 | 0 | 0 | 0.12 | 0 |
| 26.62 | Selina-3,7(11)-diene | 1545 | 1545 | 0 | 0 | 0 | 0 | 0 | 0 | 0.09 | 0 | 0 | 0.09 | 0 | 0 |
| 27.77 | β-Caryophyllene oxide | 1580 | 1582 | 0.15 | 0 | 0 | 0 | 0 | 0 | 0 | 0 | 0 | 0 | 0 | 0 |
| 28.64 | 1-*epi*-Cubenol | 1631 | 1627 | 0 | 0.34 | 0.58 | 0 | 0.20 | 0 | 0 | 0 | 0 | 0 | 0 | 0.18 |
| 29.12 | Xylose isomer 1, 4TMS | 1641 | 1644 | 0 | 0 | 0 | 0 | 0.35 | 0 | 0 | 0 | 0 | 0 | 0 | 0 |
| 30.91 | Xylose isomer 2, 4TMS | 1650 | 1652 | 0 | 0.32 | 0 | 0 | 0 | 0 | 0 | 0 | 0 | 0 | 0 | 0 |
| 31.18 | Arabinose isomer 1, TMS | 1669 | 1666 | 0 | 0.36 | 0 | 0 | 0 | 0 | 0 | 0 | 0 | 0 | 0.12 | 0 |
| 31.47 | Arabinose isomer 2, TMS | 1672 | 1674 | 0 | 0 | 0 | 0 | 0.22 | 0 | 0 | 0 | 0 | 0 | 0 | 0 |
| 31.93 | Ribonic acid, 5TMS | 1773 | 1769 | 0.93 | 0.37 | 0.97 | 0.78 | 0.60 | 0.59 | 0 | 0 | 0 | 0 | 0 | 0 |
| 32.56 | Fructose isomer 1, 5TMS | 1796 | 1792 | 1.85 | 2.46 | 0 | 1.68 | 1.50 | 1.49 | 0.42 | 0 | 0.11 | 0.22 | 0.48 | 0.18 |
| 32.73 | Fructose isomer 2, 5TMS | 1803 | 1799 | 0 | 1.39 | 0 | 2.53 | 2.85 | 2.51 | 0 | 0 | 0 | 0 | 1.32 | 0 |
| 32.81 | Shikimic acid, 4TMS | 1816 | 1820 | 22.79 | 15.14 | 16.73 | 8.16 | 14.13 | 6.11 | 21.27 | 0.66 | 26.67 | 32.15 | 23.01 | 18.42 |
| 32.94 | Galactose isomer 1, 6TMS | 1834 | 1836 | 0 | 0 | 0 | 0 | 0 | 0 | 4.58 | 0 | 0 | 0 | 0 | 0 |
| 33.15 | Glucose isomer 1, 6TMS | 1837 | 1839 | 0 | 0 | 0 | 1.51 | 0 | 0 | 0 | 0 | 0 | 0 | 0 | 0 |
| 33.16 | Galactose isomer 2, 6TMS | 1840 | 1842 | 1.26 | 0 | 0 | 0 | 0 | 0 | 0 | 0 | 0.57 | 0.61 | 0.70 | 0.50 |
| 33.28 | Glucose isomer 2, 6TMS | 1844 | 1844 | 0.90 | 0.47 | 0.56 | 1.18 | 0.39 | 1.39 | 0.27 | 0 | 0 | 0 | 0 | 0 |
| 33.40 | Mannose isomer 1, 6TMS | 1848 | 1847 | 0.57 | 0 | 0 | 0 | 0 | 3.33 | 0 | 0 | 0 | 0 | 0 | 0 |
| 33.44 | Mannose isomer 2, 6TMS | 1851 | 1849 | 0.26 | 0.92 | 0.88 | 3.70 | 1.19 | 0 | 0.28 | 0 | 0.11 | 0.14 | 0.25 | 0.13 |
| 33.53 | Myristic acid, 1TMS | 1853 | 1855 | 0 | 0 | 0 | 12.44 | 0 | 11.09 | 0 | 0 | 0 | 0 | 0 | 0 |
| 33.68 | (+)-Quinic acid, 5TMS | 1854 | 1859 | 13.41 | 3.79 | 5.72 | 18.55 | 15.90 | 17.92 | 0 | 0 | 0.81 | 0.67 | 0.52 | 2.31 |
| 33.78 | Sorbose isomer 1, 5TMS | 1866 | 1864 | 0.27 | 0 | 0 | 0 | 0 | 0 | 2.38 | 0 | 0 | 0 | 0 | 0 |
| 33.92 | Sorbose isomer 2, 5TMS | 1872 | 1873 | 0 | 0 | 0 | 1.65 | 0 | 0 | 0 | 0 | 0 | 0.36 | 0 | 0 |

| | | | | | | | | | | | | | | | |
|---|---|---|---|---|---|---|---|---|---|---|---|---|---|---|---|
| 34.34 | 1-Methyl-α-D-glucopyranoside, 4TMS | 1877 | 1882 | 0.52 | 0.41 | 0.42 | 0.58 | 0.40 | 0.44 | 0 | 0 | 0 | 0 | 0.13 | 0 |
| 34.72 | 2-Ketogluconic acid methoxyamine, 5TMS | 1896 | 1893 | 0.12 | 0 | 0 | 0.32 | 0.23 | 0 | 0 | 0 | 0 | 0 | 0 | 0 |
| 35.64 | Gallic acid, 4TMS | 1972 | 1978 | 2.34 | 2.36 | 7.69 | 8.00 | 2.54 | 3.82 | 5.61 | 0.24 | 1.22 | 5.35 | 2.64 | 0.35 |
| 36.13 | β-D-Glucopyranose, 5TMS | 1978 | 1985 | 0.43 | 0.43 | 0.51 | 0.49 | 0.54 | 0.54 | 0.17 | 0 | 0 | 0 | 0.12 | 0 |
| 37.33 | Palmitic Acid, 1TMS | 2041 | 2040 | 0.49 | 1.15 | 0.64 | 2.68 | 0.81 | 1.90 | 0.48 | 0.36 | 0.16 | 0.17 | 0 | 0.13 |
| 38.18 | Methyl linoleate | 2094 | 2096 | 0 | 0 | 0 | 0 | 0 | 0 | 0 | 0 | 0.08 | 0 | 0 | 0 |
| 39.65 | Phytol, 1TMS | 2162 | 2168 | 2.04 | 1.50 | 0.85 | 0.48 | 0.72 | 1.07 | 0 | 0 | 0 | 0 | 0 | 0 |
| 40.40 | Oleic Acid, 1TMS | 2220 | 2222 | 0 | 0 | 0 | 0 | 0 | 0 | 0.11 | 0 | 0 | 0 | 0 | 0 |
| 40.89 | Stearic acid, 1TMS | 2239 | 2240 | 0 | 0 | 0 | 0.34 | 0 | 0 | 0.22 | 0 | 0 | 0 | 0 | 0 |
| 44.76 | Ginkgol, 1TMS | 2476 | 2475 | 0.25 | 0 | 0 | 0 | 0 | 0 | 0 | 0.17 | 0 | 0 | 0 | 0 |
| 45.11 | Pentacosane | 2501 | 2500 | 0 | 0.30 | 0 | 0.31 | 0 | 0.32 | 0 | 0 | 0 | 0 | 0 | 0 |
| 45.93 | 2-Palmitoylglycerol, 2TMS | 2552 | 2560 | 0.26 | 0.37 | 0.54 | 0.44 | 0.49 | 0.49 | 0.11 | 0.14 | 0 | 0 | 0.08 | 0 |
| 46.41 | 1-Monopalmitin, 2TMS | 2583 | 2580 | 2.19 | 2.44 | 3.46 | 1.86 | 2.58 | 2.87 | 0.85 | 0.82 | 0.52 | 0.51 | 0.79 | 0.48 |
| 46.85 | Sucrose isomer 1, 8TMS | 2623 | 2620 | 0 | 0 | 0.35 | 0 | 0 | 0 | 0 | 0 | 0 | 0 | 0 | 0 |
| 47.13 | Sucrose isomer 2, 8TMS | 2651 | 2650 | 0 | 0.24 | 0 | 0 | 0 | 0 | 0 | 0 | 0 | 0 | 0 | 0 |
| 47.83 | Cardanol C17:1. 1TMS | 2670 | 2674 | 0.22 | 0 | 0 | 0 | 0 | 0 | 0 | 0 | 0 | 0 | 0 | 0 |
| 48.10 | *n*-Heptacosane | 2697 | 2700 | 0.27 | 0.97 | 0.37 | 0.65 | 0.51 | 0.81 | 0 | 0.09 | 0 | 0 | 0 | 0 |
| 48.75 | 2-Monostearin, 2TMS | 2764 | 2768 | 1.32 | 0.92 | 1.90 | 1.22 | 0.96 | 1.52 | 0.37 | 0.21 | 0.23 | 0.23 | 0.27 | 0.17 |
| 49.23 | 1-Monostearin, 2TMS | 2777 | 2779 | 5.88 | 2.29 | 8.21 | 3.40 | 2.89 | 5.59 | 2.09 | 0.88 | 1.21 | 1.10 | 1.16 | 1.01 |
| 49.55 | *n*-Octacosane | 2799 | 2800 | 2.25 | 0 | 0 | 1.19 | 0 | 0.60 | 0 | 1.96 | 0 | 0 | 0 | 0 |
| 49.66 | *trans*-Squalene | 2808 | 2810 | 1.51 | 1.35 | 0.61 | 0.59 | 0.59 | 0.32 | 0.19 | 0.14 | 0 | 0 | 0 | 0.07 |
| 49.93 | Ginkgolic acid 15:1, 2TMS | 2827 | 2824 | 0 | 0 | 0 | 0 | 0 | 0 | 1.51 | 1.06 | 0 | 0 | 0 | 0 |
| 50.03 | Hydroginkgolic acid | 2838 | 2839 | 0.13 | 0 | 0 | 0 | 0 | 0 | 0.48 | 0.77 | 0 | 0 | 0 | 0 |
| 50.60 | Catechin | 2870 | 2869 | 0.25 | 0.58 | 5.77 | 0 | 0.65 | 1.83 | 1.06 | 0.10 | 1.01 | 0.97 | 2.67 | 0.75 |
| 50.88 | Nonacosane | 2902 | 2900 | 0.58 | 1.05 | 0.32 | 1.11 | 1.67 | 0.93 | 0 | 0.22 | 0 | 0 | 0 | 0 |
| 50.99 | 5-*O*-Coumaroyl-D-quinic acid | 2910 | 2916 | 0 | 0 | 0 | 0 | 0 | 0 | 11.41 | 0 | 9.65 | 34.43 | 36.36 | 16.46 |
| 51.15 | Epigallocatechin | 2923 | 2927 | 0.61 | 1.32 | 1.91 | 0.24 | 0.74 | 0.50 | 6.09 | 0.13 | 16.63 | 2.38 | 4.91 | 0.74 |
| 51.34 | Unidentified compound 1 | 2932 | | 0 | 0 | 0 | 0 | 0 | 0 | 0.09 | 0 | 0 | 0.14 | 0.30 | 0 |
| 51.45 | 1-Hexacosanol, 1TMS | 2943 | 2945 | 0.60 | 0.57 | 0.98 | 0.71 | 1.24 | 1.30 | 0.10 | 0.09 | 0.11 | 0.31 | 0.48 | 0.22 |
| 51.55 | Galactinol isomer 1, 9TMS | 2948 | 2950 | 0 | 0 | 0 | 0 | 0 | 0 | 0 | 0 | 0 | 0.19 | 0.35 | 0.28 |

| | | | | | | | | | | | | | | | |
|---|---|---|---|---|---|---|---|---|---|---|---|---|---|---|---|
| 51.81 | Galactinol isomer 2, 9TMS | 2970 | 2972 | 0.48 | 1.38 | 1.08 | 1.00 | 1.42 | 1.08 | 0.26 | 0.56 | 0.39 | 0 | 0.64 | 0.35 |
| 51.97 | Unidentified compound 2 | 2985 | | 0 | 0 | 0 | 0 | 0 | 0 | 0 | 0.37 | 0 | 0 | 0.26 | 0 |
| 52.38 | Unidentified compound 3 | 3012 | | 0.86 | 0 | 0 | 0.39 | 0 | 0.26 | 0 | 0.20 | 0 | 0 | 0 | 0 |
| 52.60 | Ginkgolic acid 17:1 isomer 1, 2TMS | 3023 | 3025 | 0 | 0 | 0 | 0 | 0 | 0 | 0.20 | 0 | 0 | 0 | 0 | 0 |
| 52.67 | Ginkgolic acid 17:1 isomer 2, 2TMS | 3029 | | 0 | 0 | 0 | 0 | 0 | 0 | 0.22 | 0 | 0 | 0 | 0 | 0 |
| 52.75 | Unidentified compound 4 | 3038 | | 0.48 | 1.52 | 0.67 | 0.59 | 0.67 | 0.50 | 0 | 0.18 | 0 | 0 | 0 | 0 |
| 53.50 | α-Tocopherol, 1TMS | 3099 | 3101 | 0.32 | 0.24 | 0 | 0.19 | 0.24 | 0 | 0 | 0 | 0 | 0 | 0 | 0 |
| 53.87 | γ-Tocopherol, 1TMS (Vitamin E) | 3130 | 3126 | 13.58 | 19.98 | 16.43 | 5.75 | 11.11 | 8.99 | 0.17 | 0.64 | 0.13 | 0 | 0 | 0.08 |
| 53.99 | *trans*-3-*O*-Caffeoyl-D-quinic acid, 6TMS | 3139 | 3140 | 1.02 | 0.57 | 0.98 | 0.81 | 1.23 | 1.55 | 0.17 | 0.13 | 0 | 0 | 0.08 | 0 |
| 55.20 | Campesterol, 1TMS | 3234 | 3240 | 0.74 | 0.86 | 0.36 | 0.49 | 0.84 | 0.37 | 0.31 | 1.01 | 0.10 | 0.12 | 0.24 | 0.34 |
| 55.30 | Unidentified compound 5 | 3245 | | 0.78 | 1.27 | 0 | 0.45 | 0.75 | 0 | 0 | 0.18 | 0 | 0 | 0 | 0.08 |
| 55.52 | 3β-Lanosta-9(11),24-dien-3-ol | 3260 | 3265 | 0 | 0 | 0 | 0 | 0 | 0 | 0.66 | 1.55 | 0.66 | 0.76 | 0.68 | 1.30 |
| 55.77 | Lanosta-8,24-dien-3-one | 3279 | 3286 | 0 | 0 | 0 | 0 | 0 | 0 | 0.09 | 0.16 | 0 | 0.10 | 0.15 | 0.17 |
| 56.14 | Lanosterol, 1TMS | 3311 | 3314 | 0 | 1.01 | 0.35 | 0 | 0.95 | 0 | 2.45 | 4.87 | 2.38 | 5.90 | 5.85 | 9.53 |
| 56.24 | Lanosta-7,9,24-trien-3β-ol, 1TMS | 3317 | 3321 | 0 | 0 | 0 | 0 | 0 | 0 | 0 | 0 | 0 | 0.17 | 0.20 | 0.31 |
| 56.35 | β-Sitosterol | 3322 | 3328 | 5.95 | 5.27 | 4.96 | 3.50 | 5.67 | 5.78 | 1.06 | 3.92 | 0.83 | 0.44 | 0.72 | 0.99 |
| 56.44 | Stigmasterol, 1TMS | 3331 | 3333 | 0 | 0 | 0 | 0 | 0.43 | 0 | 0.22 | 0 | 0.24 | 0.17 | 0.26 | 0.43 |
| 56.53 | 1-Triacontanol | 3337 | 3340 | 0.82 | 0 | 0.28 | 0.26 | 0.71 | 0.58 | 0.09 | 0 | 0 | 0 | 0 | 0 |
| 56.62 | β-Amyrin | 3344 | 3351 | 1.49 | 3.23 | 1.43 | 1.02 | 2.10 | 1.55 | 1.14 | 1.14 | 0.61 | 0.52 | 0.94 | 1.91 |
| 56.74 | 3-*O*-Acetyl-6-methoxy-cycloartenol | 3350 | 3355 | 0 | 1.58 | 0.92 | 0 | 2.03 | 0.86 | 0.89 | 3.45 | 1.42 | 0.98 | 1.89 | 2.68 |
| 56.80 | Stigmastan-3-ol, 1TMS | 3357 | 3360 | 0 | 0.56 | 0 | 0 | 1.37 | 0 | 0.26 | 0 | 0 | 0 | 0 | 0 |
| 57.22 | α-Amyrin, 1TMS | 3381 | 3390 | 0 | 0.53 | 2.80 | 1.79 | 1.13 | 3.35 | 0.10 | 0.45 | 0 | 0 | 0 | 0.08 |
| 57.33 | Lupeol, 1TMS | 3393 | 3399 | 7.70 | 10.55 | 4.03 | 3.41 | 7.07 | 3.82 | 0.55 | 0.99 | 0.45 | 0.56 | 0.82 | 1.42 |
| 57.77 | Unidentified compound 6 | 3416 | | 0 | 0 | 0 | 0.84 | 0 | 0 | 0.16 | 2.67 | 0.08 | 0 | 0 | 0 |
| 57.96 | Unidentified compound 7 | 3425 | | 0 | 0 | 0 | 0 | 0 | 0 | 0.20 | 2.24 | 0.20 | 0 | 0.16 | 0.29 |
| 58.52 | 9,19-Cyclolanostan-3-ol, 24-methylene-, (3β)-, O-TMS | 3460 | 3463 | 0 | 0.52 | 0.33 | 0 | 0.51 | 0 | 1.79 | 1.64 | 1.54 | 0.70 | 1.13 | 3.80 |
| 58.88 | Erythrodiol, 1TMS | 3488 | 3494 | 0 | 0.54 | 0 | 0 | 0.63 | 0 | 1.13 | 1.59 | 0.94 | 0.40 | 0.65 | 1.90 |
| 59.43 | Oleanolic acid, 2TMS | 3524 | 3520 | 0 | 0 | 0 | 0 | 0 | 0 | 0.58 | 0 | 0.44 | 0 | 0 | 0.33 |
| 59.56 | Ursolic acid, 2TMS | 3535 | 3539 | 0 | 0 | 0.71 | 0 | 0.69 | 0.70 | 0 | 22.47 | 0 | 0.57 | 0.57 | 5.50 |
| 59.66 | Masticadienonic acid | 3540 | | 0 | 0 | 0 | 0 | 0 | 0 | 0 | 0 | 11.33 | 0 | 0 | 0 |

| | | | | | | | | | | | | | | | |
|---|---|---|---|---|---|---|---|---|---|---|---|---|---|---|---|
| 59.72 | Uvaol, 1TMS | 3550 | 3552 | 0 | 0 | 0 | 0 | 0 | 0 | 6.53 | 0.18 | 0.14 | 0 | 0.14 | 0.27 |
| 59.88 | Ursolic aldehyde | 3556 | 3554 | 0.89 | 2.35 | 0.88 | 0 | 2.55 | 0.78 | 0 | 4.46 | 3.75 | 0.77 | 1.15 | 4.98 |
| 60.06 | (3β)-Lup-20(29)-ene-3,28-diol | 3560 | 3563 | 0 | 0 | 0 | 0 | 0 | 0 | 3.28 | 1.10 | 0 | 0 | 0 | 0 |
| 60.32 | (3β)-Lup-20(29)-en-28-al, 3TMS | 3568 | 3570 | 0 | 0 | 0.41 | 0 | 0 | 0 | 0.33 | 5.23 | 0 | 0 | 0.76 | 0 |
| 60.47 | Betulinic acid, 2TMS | 3581 | 3586 | 0 | 2.06 | 0 | 0 | 1.39 | 0 | 1.85 | 2.58 | 1.78 | 0.45 | 0 | 3.13 |
| 60.96 | Unidentified compound 8 | 3603 | | 0 | 0 | 0 | 0 | 0 | 0 | 0.18 | 0.33 | 0 | 0.10 | 0 | 0.31 |
| 61.20 | Betulinaldehyde | 3615 | 3618 | 0 | 0 | 0 | 0 | 0 | 0 | 0.44 | 0.69 | 0.32 | 0.31 | 0.28 | 0.85 |
| 61.63 | Unidentified compound 9 | 3640 | | 0 | 0 | 0 | 0 | 0 | 0 | 0.15 | 0.24 | 0.12 | 0 | 0 | 0.16 |
| 62.49 | Unidentified compound 10 | 3691 | | 0 | 0 | 0 | 0 | 0 | 0 | 0 | 6.75 | 0.41 | 0 | 0.18 | 0.39 |
| 62.80 | Neurosporaxanthin methyl ester | 3712 | 3717 | 0 | 0 | 0 | 0 | 0 | 0 | 0 | 7.75 | 3.37 | 1.08 | 0.58 | 3.63 |
| 62.99 | Tetratriacontanol, 1TMS | 3724 | 3722 | 0 | 0 | 0 | 0 | 0 | 0 | 3.88 | 0 | 0 | 0 | 0 | 0 |
| 64.80 | Pentatriacontanol, 1TMS | 3827 | 3824 | 0 | 0 | 0 | 0 | 0 | 0 | 0 | 4.91 | 3.14 | 0.69 | 0.30 | 3.89 |
| 65.05 | Unidentified compound 11 | 3841 | | 0 | 0 | 0 | 0 | 0 | 0 | 4.68 | 0 | 0 | 0 | 0 | 0 |

*RT – retention time, min; **RIcalc – relative retention index, calculated, using a standard mixture of aliphatic hydrocarbons (C10–C40, Sigma) under the same temperature program; ***RIlit - RIs of authentic standards [18-20]; ****All values are expressed in Total Ion Current (TIC) %.

**Table S2.** GC/MS profiling of compounds in MeOH extracts of *Pistacia palaestina* leaves and galls induced by *Paracletus* (P), *Baizongia* (B), and *Geoica* (G) across three trees (T1, T2, and T3).

| RT* | Name | $RI_{calc}$** | $RI_{lit}$*** | T1L | T2L | T3L | T1P | T2P | T3P | T1G | T2G | T3G | T1B | T2B | T3B |
|---|---|---|---|---|---|---|---|---|---|---|---|---|---|---|---|
| 13.58 | Lactic acid, 2TMS | 1062 | 1066 | 0.14**** | 0 | 0 | 0 | 0 | 0 | 0 | 0 | 0 | 0 | 0 | 0 |
| 17.00 | 3-Hydroxyisovaleric acid, 2TMS | 1186 | 1204 | 0.10 | 0 | 0 | 0 | 0 | 0 | 0 | 0 | 0 | 0 | 0 | 0 |
| 19.34 | Dimethyl malate, 1TMS | 1245 | 1248 | 0.16 | 0 | 0 | 0.25 | 0 | 0.28 | 0 | 0 | 0 | 0 | 0.75 | 0 |
| 19.88 | Glycerol, 3TMS | 1264 | 1265 | 1.30 | 0.47 | 1.37 | 2.34 | 0.93 | 2.19 | 1.51 | 0.85 | 0.87 | 0.98 | 0.40 | 5.98 |
| 21.57 | Heptyl amine, 1TMS | 1336 | 1337 | 3.69 | 0 | 0 | 0 | 0 | 0 | 0 | 0 | 0 | 1.21 | 0 | 0 |
| 25.45 | Malic acid, 3TMS | 1483 | 1480 | 0 | 0 | 0 | 0.13 | 0.14 | 0.13 | 0.11 | 0 | 0.13 | 0 | 0.12 | 0 |
| 26.55 | Pyrogallol, 3TMS | 1534 | 1537 | 0.13 | 0 | 0 | 0.15 | 0.16 | 0.14 | 0.14 | 0 | 0.17 | 0.09 | 0.19 | 0 |
| 26.93 | Methyl β-D-ribofuranoside, 3TMS | 1548 | 1552 | 0.08 | 0 | 0 | 0 | 0.13 | 0.15 | 0 | 0 | 0 | 0 | 0 | 0 |
| 27.48 | 1-Dodecanol, 1TMS | 1571 | 1572 | 0.35 | 0.40 | 0.24 | 0.09 | 0.24 | 0.11 | 0 | 0 | 0 | 0 | 0 | 0 |
| 27.61 | Methyl xylopyranoside, 3TMS isomer | 1577 | 1580 | 0.91 | 0.88 | 1.48 | 0.74 | 0.93 | 1.13 | 0 | 0 | 0 | 0.09 | 0.17 | 0 |
| 27.80 | Methyl xylopyranoside, 3TMS isomer | 1584 | 1586 | 0.16 | 0.20 | 0.23 | 0.13 | 0.18 | 0.23 | 0 | 0 | 0 | 0 | 0 | 0 |
| 28.71 | *L*-Rhamnose, 4TMS | 1655 | 1657 | 0.08 | 0.53 | 0 | 0.55 | 0.85 | 0.42 | 0 | 0 | 0 | 0 | 0.77 | 0 |
| 29.38 | 4-Hydroxybenzoic acid, 2TMS | 1669 | 1670 | 0 | 0.17 | 0 | 0 | 0 | 0 | 0 | 0 | 0 | 0 | 0 | 0 |
| 30.14 | Ribose, 4TMS | 1687 | 1688 | 0 | 0.29 | 0 | 0.22 | 0.17 | 0.14 | 0 | 0 | 0 | 0 | 0 | 0 |
| 30.26 | Xylitol, 5TMS | 1696 | 1701 | 0 | 0 | 0 | 0 | 0 | 0 | 0 | 0 | 0 | 0.09 | 0.11 | 0 |
| 30.35 | *n*-Heptadecane | 1700 | 1700 | 0.09 | 0 | 0 | 0.11 | 0.18 | 0.13 | 0.11 | 0 | 0.13 | 0 | 0.16 | 0 |
| 31.11 | *L*-Glycerol-2-phosphate, 4TMS | 1732 | 1735 | 0.10 | 0 | 0 | 0 | 0 | 0 | 0 | 0 | 0 | 0.08 | 0 | 0 |
| 31.30 | 1-Heptadecanol, 1 TMS | 1741 | 1744 | 0.09 | 0 | 0 | 0 | 0.22 | 0.09 | 0.13 | 0 | 0 | 0 | 0.16 | 0 |
| 31.44 | Pentonic acid-1,4-lactone, 3TMS | 1750 | 1752 | 0 | 0.78 | 0.29 | 0.26 | 0.88 | 0 | 0.45 | 0 | 0 | 0 | 0.43 | 0 |
| 31.50 | Ribonic acid, 5TMS | 1754 | 1756 | 0.32 | 0 | 0 | 0.31 | 0.37 | 0.50 | 0.59 | 0 | 0.77 | 0.49 | 0.41 | 1.42 |
| 31.54 | Pentonic acid, 5TMS | 1757 | 1761 | 0.19 | 0 | 0 | 0 | 0 | 0 | 0 | 0 | 0 | 0 | 0 | 0 |
| 31.70 | *n*-Octadecane | 1796 | 1800 | 0 | 0.20 | 0 | 0.11 | 0 | 0.12 | 0 | 0 | 0 | 0 | 0 | 0 |
| 31.80 | Shikimic acid, 4TMS | 1815 | 1818 | 12.24 | 9.59 | 8.40 | 6.54 | 6.04 | 7.85 | 14.56 | 8.25 | 14.23 | 12.60 | 11.36 | 22.66 |
| 32.02 | (-)-Quinic acid, 5TMS | 1835 | 1839 | 35.22 | 23.84 | 38.66 | 33.08 | 32.46 | 35.80 | 11.24 | 13.10 | 30.43 | 16.04 | 19.91 | 29.27 |
| 32.09 | DL-2-Methylcitric acid, TMS | 1842 | 1846 | 0.43 | 0 | 0.74 | 0.62 | 1.83 | 0.43 | 0 | 0 | 0.33 | 0.23 | 0.27 | 0 |
| 32.24 | 2-Keto-*L*-gluconic acid, 5TMS | 1860 | 1858 | 0.15 | 0.31 | 0 | 0 | 0 | 0 | 0.23 | 0 | 0 | 0.43 | 0.76 | 0.63 |
| 32.31 | 1-Methyl-α-D-glucopyranoside, 4TMS isomer | 1868 | 1869 | 0.27 | 0.61 | 0 | 0 | 0 | 0 | 0 | 0 | 0 | 0 | 0 | 0 |
| 32.46 | Fructose, 5TMS isomer | 1877 | 1874 | 0 | 0.19 | 0 | 0 | 0 | 0 | 0 | 0 | 0 | 0.11 | 0.20 | 0 |

| | | | | | | | | | | | | | | | |
|---|---|---|---|---|---|---|---|---|---|---|---|---|---|---|---|
| 32.56 | Galactose, 6TMS isomer | 1884 | 1883 | 0.83 | 1.53 | 0.67 | 1.18 | 1.66 | 0.55 | 0.32 | 0.62 | 0.23 | 0.84 | 1.75 | 0.98 |
| 32.68 | Glucose, 6TMS isomer | 1895 | 1892 | 0.12 | 0 | 0 | 0 | 0 | 0 | 0 | 0 | 0 | 0.20 | 0 | 0 |
| 32.74 | 1-Methyl-$\alpha$-D-glucopyranoside, 4TMS isomer | 1901 | 1898 | 0 | 0 | 2.25 | 0 | 0 | 0 | 0 | 0 | 0 | 0 | 0 | 0 |
| 32.79 | Methyl galactoside, 4TMS isomer | 1911 | 1907 | 4.57 | 12.03 | 3.17 | 9.13 | 9.42 | 2.93 | 3.87 | 7.41 | 1.18 | 12.83 | 16.08 | 9.30 |
| 32.92 | Methyl galactoside, 4TMS isomer | 1915 | 1916 | 1.45 | 0.94 | 1.50 | 1.57 | 1.27 | 1.49 | 0.12 | 0 | 0.15 | 0.61 | 0.71 | 0 |
| 33.07 | Fructose, 5TMS isomer | 1920 | 1922 | 0.19 | 0 | 0 | 0.34 | 0 | 0 | 0 | 0 | 0 | 0.22 | 0 | 0 |
| 33.17 | Galactose, 6TMS isomer | 1924 | 1925 | 0.22 | 0 | 0 | 0 | 0 | 0 | 0 | 0 | 0 | 0.44 | 0.88 | 0 |
| 33.42 | Glucose, 6TMS isomer | 1932 | 1930 | 0 | 0.59 | 0 | 0.60 | 1.38 | 0.41 | 0 | 0 | 0 | 0.16 | 0.64 | 0 |
| 33.46 | Glucitol, 6TMS | 1936 | 1934 | 0 | 0 | 0 | 0 | 0 | 0 | 0 | 0 | 0 | 0.13 | 0 | 0 |
| 33.56 | Galactitol, 6TMS | 1939 | 1941 | 0.48 | 0.29 | 0.38 | 0.41 | 0.29 | 0.39 | 0 | 0 | 0 | 0.14 | 0 | 0 |
| 33.68 | *trans-p*-Coumaric acid, 2TMS | 1947 | 1946 | 3.94 | 0.76 | 0 | 1.41 | 0.49 | 3.56 | 0 | 0 | 0.21 | 3.06 | 0.54 | 0.55 |
| 33.81 | Methyl glucoside, 4TMS isomer | 1954 | 1952 | 0 | 0 | 0 | 0 | 0 | 0 | 0.36 | 0 | 0.56 | 0.22 | 0 | 0 |
| 34.13 | Methyl glucoside, 4TMS isomer | 1965 | 1961 | 8.01 | 10.37 | 14.31 | 7.83 | 7.53 | 11.17 | 7.99 | 15.07 | 5.33 | 7.29 | 5.34 | 6.77 |
| 34.24 | 4-*O*-Methyl-myo-inositol, 5TMS | 1970 | 1968 | 2.18 | 1.70 | 7.80 | 3.42 | 2.68 | 5.06 | 8.57 | 11.72 | 13.03 | 1.06 | 1.81 | 2.40 |
| 34.30 | Caffeic acid, 3TMS | 1973 | 1975 | 0 | 0.37 | 0 | 0 | 0 | 0 | 0 | 0 | 0 | 0.28 | 0.21 | 0 |
| 34.35 | Galactonic acid, 6TMS | 1979 | 1980 | 1.53 | 9.24 | 2.07 | 6.71 | 7.96 | 3.26 | 0.77 | 2.10 | 0.21 | 3.11 | 4.47 | 2.78 |
| 34.48 | Gluconic acid, 6TMS | 1986 | 1984 | 4.59 | 3.32 | 5.89 | 3.28 | 2.81 | 2.54 | 1.62 | 2.40 | 1.23 | 5.30 | 2.35 | 3.20 |
| 34.55 | Gallic acid, 4TMS | 1994 | 1997 | 0.15 | 0.42 | 0 | 0.49 | 0.44 | 0.31 | 0 | 0 | 0 | 0 | 0 | 0 |
| 34.66 | Hexonic acid, 6TMS | 2003 | 2006 | 0 | 0 | 0 | 0 | 0 | 0 | 0 | 0 | 0 | 0.12 | 0 | 0 |
| 34.76 | Coniferylalcohol, 1TMS | 2008 | 2009 | 0.11 | 0 | 0 | 0.20 | 0.28 | 0.11 | 0.09 | 0 | 0.09 | 0.16 | 0.10 | 0 |
| 35.07 | Methyl heptanoate | 2023 | 2020 | 0.79 | 0.61 | 0.64 | 1.16 | 0.96 | 1.34 | 0.35 | 0 | 0 | 1.67 | 0.67 | 0 |
| 35.64 | Palmitic acid, 1TMS | 2041 | 2046 | 1.40 | 0.58 | 0.39 | 1.36 | 0.86 | 2.30 | 2.71 | 3.97 | 0.92 | 1.33 | 1.11 | 0.66 |
| 36.13 | Myo-Inositol, 6TMS | 2075 | 2080 | 1.15 | 10.43 | 1.77 | 6.54 | 7.90 | 3.47 | 0.61 | 1.93 | 0.13 | 2.86 | 4.60 | 2.57 |
| 38.07 | Methyl linolenate | 2094 | 2098 | 1.55 | 3.80 | 4.06 | 1.27 | 1.67 | 1.79 | 4.77 | 5.91 | 2.70 | 4.36 | 3.23 | 6.61 |
| 38.22 | *n*-Eicosane | 2100 | 2100 | 0.12 | 0 | 0 | 0.13 | 0.18 | 0.19 | 0.43 | 0 | 0.27 | 0.72 | 0.46 | 0 |
| 38.33 | 3-Deoxy-arabino-hexaric acid, 5TMS | 2111 | 2113 | 1.33 | 0.66 | 0.82 | 0.52 | 0.87 | 1.23 | 0.22 | 0 | 0.15 | 0.48 | 0.38 | 0 |
| 38.85 | Methyl stearate | 2123 | 2126 | 0.33 | 0 | 0.33 | 0.24 | 0.20 | 0.40 | 0.21 | 0 | 0 | 0.31 | 0.11 | 0 |
| 41.64 | Glucose 6-phosphate, 6TMS | 2280 | 2283 | 0.09 | 0 | 0 | 0.39 | 0 | 0.24 | 0 | 0 | 0 | 0.15 | 0 | 0 |
| 42.08 | 2-*O*-Glycerol-$\alpha$-D-galactopyranoside | 2309 | 2313 | 0.17 | 0 | 0 | 0.08 | 0 | 0.10 | 0 | 0 | 0 | 0.08 | 0 | 0 |
| 46.41 | 2-Monopalmitin, 2TMS | 2582 | 2580 | 0.21 | 0.40 | 0.23 | 0 | 0 | 0.23 | 0 | 0 | 0 | 0.11 | 0.19 | 0 |
| 46.83 | 1-Monohexadecanoylglycerol (2TMS | 2591 | 2590 | 0.10 | 0 | 0 | 0 | 0 | 0 | 0 | 0 | 0 | 0 | 0 | 0 |

| | | | | | | | | | | | | | | | |
|---|---|---|---|---|---|---|---|---|---|---|---|---|---|---|---|
| 46.90 | Sucrose isomer, 8TMS | 2634 | 2640 | 0.42 | 0.28 | 0 | 0.40 | 0.41 | 0 | 0.23 | 0 | 0 | 0.90 | 0.44 | 0 |
| 47.20 | Sucrose isomer, 8TMS | 2651 | 2650 | 1.08 | 0.32 | 0.35 | 0.33 | 0.27 | 0.35 | 0 | 0 | 0 | 0.37 | 0.17 | 0 |
| 47.23 | Disaccharide 1 | 2639 | | 0 | 0.22 | 0 | 0 | 0 | 0 | 0 | 0 | 0 | 0 | 0 | 0 |
| 47.36 | Disaccharide 2 | 2647 | | 1.02 | 0.61 | 0.28 | 0.80 | 0 | 0.23 | 0.41 | 0.39 | 0 | 2.15 | 0.65 | 0 |
| 47.47 | Disaccharide 3 | 2654 | | 0.44 | 0 | 0 | 0.15 | 0 | 0 | 0 | 0 | 0 | 0.12 | 0 | 0 |
| 47.56 | Disaccharide 4 | 2660 | | 0.27 | 0 | 0 | 0 | 0 | 0 | 0 | 0 | 0 | 0.57 | 0 | 0 |
| 47.61 | Disaccharide 5 | 2664 | | 0.18 | 0 | 0 | 0 | 0 | 0.17 | 0 | 0 | 0 | 0 | 0 | 0 |
| 47.73 | β-D-Lactose (isomer), 8TMS | 2672 | 2680 | 0.48 | 0 | 0 | 0.25 | 0 | 0.52 | 0 | 0 | 0 | 0.33 | 0 | 0 |
| 48.02 | β-D-Lactose (isomer), 8TMS | 2692 | 2690 | 0.66 | 0 | 0 | 0.34 | 0.34 | 0.55 | 0 | 0 | 0 | 0.08 | 0 | 0 |
| 48.09 | Disaccharide 6 | 2697 | | 0.29 | 0 | 0 | 0 | 0 | 0.37 | 0 | 0 | 0 | 0.36 | 0 | 0 |
| 48.15 | Disaccharide 7 | 2700 | | 0.17 | 0 | 0 | 0 | 0 | 0 | 0 | 0 | 0 | 0.11 | 0 | 0 |
| 48.26 | Disaccharide 8 | 2708 | | 0.13 | 0 | 0 | 0 | 0 | 0 | 0 | 0 | 0 | 0.19 | 0 | 0 |
| 48.40 | Disaccharide 9 | 2718 | | 0.38 | 0 | 0 | 0.21 | 0.17 | 0.21 | 0 | 0 | 0 | 0.39 | 0 | 0 |
| 48.48 | Disaccharide 10 | 2724 | | 0.10 | 0 | 0 | 0 | 0 | 0 | 0 | 0 | 0 | 0.13 | 0 | 0 |
| 48.75 | Disaccharide 11 | 2743 | | 0 | 0 | 0 | 0 | 0 | 0 | 0 | 0 | 0 | 0.15 | 0 | 0 |
| 48.91 | Maltose, 8TMS isomer | 2755 | 2760 | 0.12 | 0 | 0 | 0 | 0 | 0 | 0 | 0 | 0 | 0 | 0 | 0 |
| 49.10 | Maltose, 8TMS isomer | 2768 | 2768 | 0.27 | 0 | 0 | 0 | 0 | 0 | 0 | 0 | 0 | 0.28 | 0 | 0 |
| 49.21 | 1-Monostearin, 2TMS | 2776 | 2773 | 0.52 | 1.61 | 0.87 | 0.16 | 0 | 0.74 | 0.11 | 0 | 0.13 | 0.46 | 0.79 | 0 |
| 49.65 | *trans*-Squalene | 2807 | 2810 | 0.10 | 0 | 0 | 0 | 0 | 0 | 0 | 0 | 0 | 0.17 | 0 | 0 |
| 49.85 | Disaccharide 12 | 2822 | | 0.08 | 0 | 0 | 0 | 0 | 0 | 0 | 0 | 0 | 0.17 | 0 | 0 |
| 50.21 | Melibiose, 8TMS isomer | 2848 | 2852 | 0.13 | 0 | 0 | 0.15 | 0 | 0.31 | 0.21 | 0 | 0.36 | 0.22 | 0.26 | 0 |
| 50.40 | Melibiose, 8TMS isomer | 2864 | 2868 | 0.76 | 0.24 | 0.53 | 2.91 | 3.97 | 3.19 | 35.36 | 26.28 | 24.65 | 9.77 | 14.72 | 4.22 |
| 50.51 | Unidentified compound 1 | 2869 | | 0 | 0 | 0 | 0 | 0 | 0 | 0.31 | 0 | 0 | 0.25 | 0 | 0 |
| 50.78 | Unidentified compound 2 | 2880 | | 0 | 0 | 0 | 0 | 0 | 0 | 0 | 0 | 0 | 0.28 | 0 | 0 |
| 50.82 | 5-*O*-Coumaroyl-D-quinic acid | 2892 | 2887 | 0.52 | 0.24 | 0.30 | 0.37 | 0.27 | 0.42 | 0.91 | 0 | 1.08 | 1.64 | 0.91 | 0 |
| 51.71 | Unidentified compound 3 | 2961 | | 0 | 0 | 0 | 0 | 0 | 0 | 0.34 | 0 | 0.30 | 0 | 0.20 | 0 |
| 56.32 | β-Sitosterol, 1TMS | 3325 | 3328 | 0 | 0 | 0 | 0 | 0 | 0 | 0 | 0 | 0 | 0.19 | 0 | 0 |

*RT – retention time, min; **RIcalc – relative retention index, calculated, using a standard mixture of aliphatic hydrocarbons (C10–C40, Sigma) under the same temperature program; ***RIlit - RIs of authentic standards [18-20]; ****All values are expressed in Total Ion Current (TIC) %.

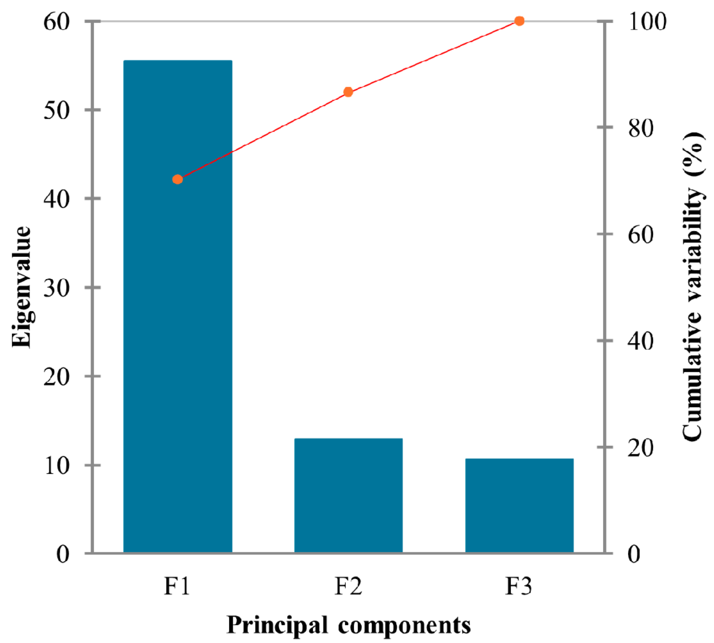


**Figure S1.** Scree plot of principal components showing explained variance for compounds in EtOAc extracts across samples of leaves and galls induced by *Paracletus*, *Baizongia*, and *Geoica* aphids.

**Table S3.** Principal components summary, eigenvalues and variance explained for compounds in EtOAc extracts across samples.

| | **F1** | **F2** | **F3** |
|---|---|---|---|
| Eigenvalue | 55.50 | 12.91 | 10.60 |
| Variability (%) | 70.25 | 16.34 | 13.42 |
| Cumulative % | 70.25 | 86.58 | 100 |

**Table S4.** PCA factor loadings highlighting variation in median TIC% of individual compounds between leaves and galls induced by *Paracletus*, *Baizongia*, and *Geoica* aphids (EtOAc extracts). Values in bold correspond to the factor for which the squared cosine is the largest.

| Compound class | Compound | F1 | Sample Association | F2 | Sample Association | F3 | Sample Association |
|---|---|---|---|---|---|---|---|
| Hydrocarbons | Pentacosane | 0.65 | | -0.17 | | **-0.74** | |
| | *n*-Heptacosane | **0.97** | *Paracletus*/Leaves | -0.09 | | -0.23 | |
| | *n*-Octacosane | 0.65 | | -0.17 | | **-0.74** | |
| | Nonacosane | **0.97** | *Paracletus*/Leaves | -0.09 | | -0.23 | |
| Lipids | Palmitic Acid | **0.93** | *Paracletus*/Leaves | 0.22 | | -0.28 | |
| | 2-Palmitoylglycerol | **0.93** | *Paracletus*/Leaves | 0.22 | | -0.28 | |
| | 1-Monopalmitin | **0.93** | *Paracletus*/Leaves | 0.22 | | -0.28 | |
| | 2-Monostearin | **0.88** | *Paracletus*/Leaves | 0.34 | | 0.34 | |
| | 1-Monostearin | **0.88** | *Paracletus*/Leaves | 0.34 | | 0.34 | |
| | α-Tocopherol | **0.91** | *Paracletus*/Leaves | 0.03 | | 0.42 | |
| | Vitamin E | **0.88** | *Paracletus*/Leaves | 0.34 | | 0.34 | |
| | 1-Hexacosanol | **0.90** | *Paracletus*/Leaves | -0.40 | | -0.16 | |
| | 1-Triacontanol | **0.97** | *Paracletus*/Leaves | -0.09 | | -0.23 | |
| | Pentatriacontanol | **-0.92** | *Geoica*/*Baizongia* | 0.36 | | -0.15 | |
| | Campesterol | **0.88** | *Paracletus*/Leaves | 0.34 | | 0.34 | |
| | β-Sitosterol | **0.93** | *Paracletus*/Leaves | 0.22 | | -0.28 | |
| | Stigmasterol | **-0.95** | *Geoica*/*Baizongia* | -0.30 | | -0.03 | |
| Carbohydrates | Ribonic acid | **0.91** | *Paracletus*/Leaves | 0.03 | | 0.42 | |
| | Fructose isomer 1 | **0.84** | *Paracletus*/Leaves | -0.28 | | 0.45 | |
| | Fructose isomer 2 | 0.65 | | -0.17 | | **-0.74** | *Paracletus* |
| | Galactose isomer 2 | -0.62 | | **-0.78** | *Geoica* | 0.09 | |
| | Glucose isomer 2 | **0.97** | *Paracletus*/Leaves | -0.09 | | -0.23 | |
| | Mannose isomer 2 | **0.90** | *Paracletus*/Leaves | -0.40 | | -0.16 | |
| | 1-Methyl-α-D-glucopyranoside | **0.97** | *Paracletus*/Leaves | -0.09 | | -0.23 | |
| | β-D-Glucopyranose | **0.97** | *Paracletus*/Leaves | -0.09 | | -0.23 | |
| | Galactinol isomer 1 | -0.62 | | **-0.78** | *Geoica* | 0.09 | |
| | Galactinol isomer 2 | **0.88** | *Paracletus*/Leaves | 0.34 | | 0.34 | |
| Organic acids | Lactic acid | **0.97** | *Paracletus*/Leaves | -0.09 | | -0.23 | |

| | | | | | | |
|---|---|---|---|---|---|---|
| | Shikimic acid | **-0.93** | *Geoica*/Leaves | -0.22 | | 0.28 |
| | (+)-Quinic acid | **0.90** | *Paracletus*/Leaves | -0.40 | | -0.16 |
| | 2-Ketogluconic acid methoxyamine | 0.65 | | -0.17 | | **-0.74** |
| Phenolics | Pyrogallol | 0.13 | | -0.35 | | **0.93** |
| | Gallic acid | 0.47 | | **-0.75** | T1P, T1B, T1G | -0.46 |
| | Catechin | **-0.84** | *Geoica*/*Baizongia* | 0.28 | | -0.45 |
| | Epigallocatechin | **-0.90** | *Geoica*/*Baizongia* | 0.40 | | 0.16 |
| | 5-*O*-Coumaroyl-D-quinic acid | **-0.95** | *Geoica*/*Baizongia* | -0.30 | | -0.03 |
| | *trans*-3-*O*-Caffeoyl-D-quinic acid | **0.93** | *Paracletus*/Leaves | 0.22 | | -0.28 |
| Terpenoids | α-Pinene | **-0.92** | *Geoica*/*Baizongia* | 0.36 | | -0.15 |
| | β-Pinene | **-0.95** | *Geoica*/*Baizongia* | -0.30 | | -0.03 |
| | α-Terpinene | -0.62 | | **-0.78** | | 0.09 |
| | β-Phellandrene | **-0.95** | *Geoica*/*Baizongia* | -0.30 | | -0.03 |
| | γ-Terpinene | **-0.95** | *Geoica*/*Baizongia* | -0.30 | | -0.03 |
| | Terpinen-4-ol | **-0.95** | *Geoica*/*Baizongia* | -0.30 | | -0.03 |
| | α-Terpineol | -0.62 | | **-0.78** | *Geoica* | 0.09 |
| | Bornyl acetate | **-0.92** | *Geoica*/*Baizongia* | 0.36 | | -0.15 |
| | β-Caryophyllene | 0.50 | | 0.13 | | **0.86** |
| | Germacrene D | 0.50 | | 0.13 | | **0.86** |
| | Zonarene | -0.62 | | **-0.78** | *Geoica* | 0.09 |
| | 1-*epi*-Cubenol | 0.50 | | 0.13 | | **0.86** |
| | Phytol | **0.91** | *Paracletus*/Leaves | 0.03 | | 0.42 |
| | *trans*-Squalene | **0.88** | *Paracletus*/Leaves | 0.34 | | 0.34 |
| | 3β-Lanosta-9(11),24-dien-3-ol | **-0.95** | *Geoica*/*Baizongia* | -0.30 | | -0.03 |
| | Lanosta-8,24-dien-3-one | **-0.95** | *Geoica*/*Baizongia* | -0.30 | | -0.03 |
| | Lanosterol | **-0.93** | *Geoica*/*Baizongia* | -0.22 | | 0.28 |
| | Lanosta-7,9,24-trien-3β-ol | -0.62 | | **-0.78** | *Geoica* | 0.09 |
| | β-Amyrin | **0.93** | *Paracletus*/Leaves | 0.22 | | -0.28 |
| | 3-*O*-Acetyl-6-methoxy-cycloartenol | **-0.93** | *Geoica*/*Baizongia* | -0.22 | | 0.28 |
| | α-Amyrin | **0.93** | *Paracletus*/Leaves | 0.22 | | -0.28 |
| | Lupeol | **0.84** | *Paracletus*/Leaves | -0.28 | | 0.45 |

| | | | | | | | |
|---|---|---|---|---|---|---|---|
| | 24-Methylenecycloartanol | **-0.90** | *Geoica*/*Baizongia* | 0.40 | | 0.16 | |
| | Erythrodiol | **-0.92** | *Geoica*/*Baizongia* | 0.36 | | -0.15 | |
| | Oleanolic acid | -0.53 | | **0.82** | | -0.20 | |
| | Ursolic acid | 0.29 | | -0.65 | | **-0.70** | *Geoica*/*Baizongia* |
| | Uvaol | **-0.92** | *Geoica*/*Baizongia* | 0.36 | | -0.15 | |
| | Ursolic aldehyde | **-0.90** | *Baizongia* | 0.40 | | 0.16 | |
| | (3β)-Lup-20(29)-en-28-al | -0.53 | | **0.82** | | -0.20 | |
| | Betulinic acid | **-0.92** | *Baizongia* | 0.36 | | -0.15 | |
| | Betulinaldehyde | **-0.92** | *Geoica*/*Baizongia* | 0.36 | | -0.15 | |
| | Neurosporaxanthin methyl ester | **-0.92** | *Geoica*/*Baizongia* | 0.36 | | -0.15 | |
| Miscellaneous | Glycerol | **0.93** | *Paracletus*/Leaves | 0.22 | | -0.28 | |
| Unidentified compounds | Unidentified compound 1 | -0.62 | | **-0.78** | | 0.09 | |
| | Unidentified compound 2 | 0.65 | | -0.17 | | **-0.74** | |
| | Unidentified compound 3 | **0.91** | | 0.03 | | 0.42 | |
| | Unidentified compound 4 | **0.91** | | 0.03 | | 0.42 | |
| | Unidentified compound 5 | -0.53 | | **0.82** | | -0.20 | |
| | Unidentified compound 6 | **-0.92** | | 0.36 | | -0.15 | |
| | Unidentified compound 7 | **-0.92** | | 0.36 | | -0.15 | |
| | Unidentified compound 8 | -0.53 | | **0.82** | | -0.20 | |
| | Unidentified compound 9 | **-0.92** | | 0.36 | | -0.15 | |

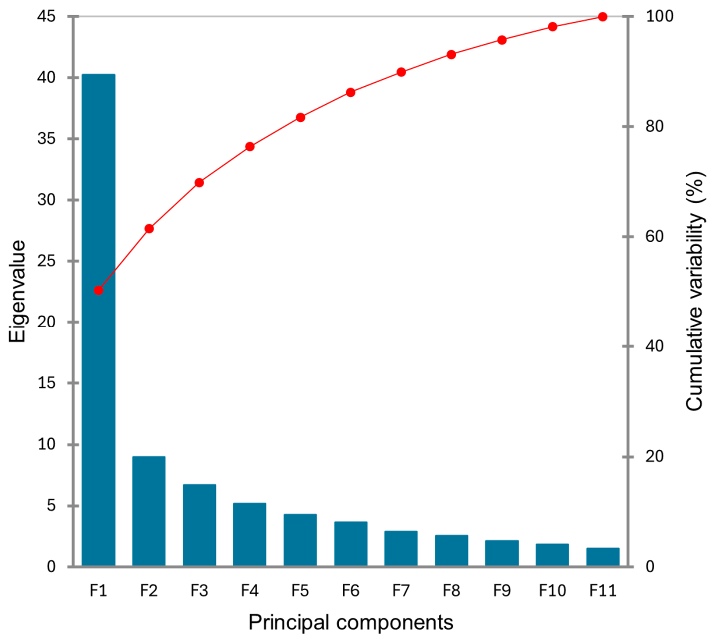


**Figure S2.** Scree plot of principal components and explained variance for compounds in EtOAc extracts of leaves and galls induced by *Paracletus*, *Baizongia*, and *Geoica* aphids across trees.

**Table S5.** Summary of principal components, eigenvalues, and variance contributions for compounds across trees (EtOAc extracts).

| | **F1** | **F2** | **F3** | **F4** | **F5** | **F6** | **F7** | **F8** | **F9** | **F10** | **F11** |
|---|---|---|---|---|---|---|---|---|---|---|---|
| Eigenvalue | 40.22 | 8.99 | 6.70 | 5.17 | 4.29 | 3.64 | 2.91 | 2.58 | 2.12 | 1.87 | 1.50 |
| Variability (%) | 50.27 | 11.23 | 8.38 | 6.47 | 5.36 | 4.56 | 3.64 | 3.22 | 2.66 | 2.33 | 1.88 |
| Cumulative % | 50.27 | 61.50 | 69.88 | 76.35 | 81.71 | 86.27 | 89.91 | 93.13 | 95.79 | 98.12 | 100 |

**Table S6.** PCA factor loadings highlighting variation in raw TIC% of individual compounds in EtOAc extracts of leaves and galls induced by *Paracletus*, *Baizongia*, and *Geoica* aphids across trees. Values in bold correspond to the factor for which the squared cosine is the largest.

| Compound class | Compound | F1 | Sample association | F2 | Sample association |
|---|---|---|---|---|---|
| Hydrocarbons | Pentacosane | **-0.59** | | 0.01 | |
| | *n*-Heptacosane | **-0.90** | T1P÷T3P; T1L÷T3L | 0.26 | |
| | *n*-Octacosane | -0.36 | | 0.31 | |
| | Nonacosane | **-0.91** | T1P÷T3P; T1L÷T3L | 0.27 | |
| Lipids | Palmitic Acid | **-0.91** | T1P÷T3P; T1L÷T3L | 0.12 | |
| | 2-Palmitoylglycerol | **-0.91** | T1P÷T3P; T1L÷T3L | 0.15 | |
| | 1-Monopalmitin | **-0.91** | T1P÷T3P; T1L÷T3L | 0.07 | |
| | 2-Monostearin | **-0.90** | T1P÷T3P; T1L÷T3L | -0.28 | |
| | 1-Monostearin | **-0.88** | T1P÷T3P; T1L÷T3L | -0.31 | |
| | α-Tocopherol | **-0.67** | T1P, T2P; T1L, T2L | 0.10 | |
| | Vitamin E | **-0.87** | T1L÷T3L; T1P÷T3P | 0.30 | |
| | 1-Hexacosanol | **-0.84** | T1P÷T3P; T1L÷T3L | -0.28 | |
| | 1-Triacontanol | **-0.78** | T1P÷T3P; T1L, T3L | -0.14 | |
| | Pentatriacontanol | **0.86** | T2B, T3B; T1G÷T3G | 0.31 | |
| | Campesterol | -0.59 | | **0.73** | T1P÷T3P; T1L÷T3L |
| | β-Sitosterol | **-0.82** | T1P÷T3P; T1L÷T3L | 0.34 | |
| | Stigmasterol | **0.60** | T1B, T3B; T1G÷T3G | -0.14 | |
| | Stigmastan-3-ol | -0.29 | | 0.22 | |
| Carbohydrates | Ribonic acid | **-0.91** | T1L÷T3L; T1P÷T3P | -0.08 | |
| | Fructose isomer 1 | **-0.61** | | -0.18 | |
| | Fructose isomer 2 | **-0.61** | | 0.01 | |
| | Galactose isomer 2 | 0.44 | | **-0.51** | T3B; T1G÷T3G |
| | Glucose isomer 2 | **-0.93** | T1P÷T3P; T1L÷T3L | -0.11 | |
| | β-D-Glucopyranose | **-0.91** | T1P÷T3P; T1L÷T3L | -0.12 | |
| | Mannose isomer 2 | **-0.57** | | -0.16 | |
| | 1-Methyl-alpha-D-glucopyranoside | **-0.90** | T1P÷T3P; T1L÷T3L | -0.18 | |
| | Galactinol isomer 1 | **0.64** | T1G÷T3G | -0.35 | |

| | | | | | |
|---|---|---|---|---|---|
| | Galactinol isomer 2 | **-0.76** | T1P÷T3P; T1L÷T3L | 0.26 | |
| Organic acids | Lactic acid | **-0.53** | | 0.33 | |
| | Shikimic acid | 0.52 | | **-0.70** | T1B, T3B; T1G÷T3G |
| | (+)-Quinic acid | **-0.82** | T1P÷T3P; T1L÷T3L | -0.12 | |
| | 2-Ketogluconic acid methoxyamine | **-0.58** | | -0.01 | |
| Phenolics | Pyrogallol | -0.09 | | 0.02 | |
| | Gallic acid | -0.42 | | **-0.60** | T1P, T1B, T1G |
| | Catechin | 0.19 | | **-0.53** | |
| | Epigallocatechin | 0.43 | | **-0.57** | T1B, T3B; T2G |
| | *trans*-3-*O*-Caffeoyl-D-quinic acid | **-0.92** | T1P÷T3P; T1L÷T3L | 0.07 | |
| | 5-*O*-Coumaroyl-D-quinic acid | **0.82** | T1B, T3B; T1G÷T3G | -0.49 | |
| Terpenoids | α-Pinene | **0.82** | T2B, T3B; T1G÷T3G | 0.15 | |
| | β-Pinene | **0.91** | T1B÷T3B; T1G÷T3G | 0.24 | |
| | α-Terpinene | **0.68** | T1G÷T3G | -0.17 | |
| | β-Phellandrene | **0.86** | T2B, T3B; T1G÷T3G | 0.01 | |
| | γ-Terpinene | **0.74** | T1B, T3B; T1G, T3G | -0.21 | |
| | Terpinen-4-ol | **0.84** | T1B, T3B; T1G÷T3G | -0.40 | |
| | α-Terpineol | **0.63** | T1G÷T3G | -0.42 | |
| | Bornyl acetate | **0.79** | T1B÷T3B; T1G, T3G | 0.40 | |
| | β-Caryophyllene | -0.34 | | 0.07 | |
| | Germacrene D | -0.34 | | 0.25 | |
| | Zonarene | **0.67** | | -0.11 | |
| | 1-*epi*-Cubenol | -0.34 | | 0.25 | |
| | Phytol | **-0.90** | T1L÷T3L; T1P÷T3P | 0 | |
| | *trans*-Squalene | **-0.88** | T1L÷T3L; T1P÷T3P | 0.19 | |
| | 3β-Lanosta-9(11),24-dien-3-ol | **0.94** | T1B÷T3B; T1G÷T3G | 0.22 | |
| | Lanosta-8,24-dien-3-one | **0.82** | T1B, T2B; T1G÷T3G | 0.22 | |
| | Lanosterol | **0.91** | T1B÷T3B; T1G÷T3G | 0.07 | |
| | Lanosta-7,9,24-trien-3β-ol | **0.66** | T1G÷T3G | -0.29 | |
| | β-Amyrin | **-0.52** | | 0.44 | |
| | 3-*O*-Acetyl-6-methoxy-cycloartenol | 0.54 | | **0.57** | |

| | | | | | |
|---|---|---|---|---|---|
| | $\alpha$-Amyrin | **-0.69** | | 0.31 | |
| | Lupeol | **-0.81** | T1P÷T3P; T1L÷T3L | 0.24 | |
| | 24-Methylenecycloartanol | **0.88** | T1B÷T3B; T1G÷T3G | 0.29 | |
| | Erythrodiol | **0.79** | T1B÷T3B; T1G÷T3G | 0.47 | |
| | Oleanolic acid | **0.56** | | -0.02 | |
| | Ursolic acid | 0.20 | | 0.45 | |
| | Uvaol | **0.77** | T1B÷T3B; T2G, T3G | 0.29 | |
| | Ursolic aldehyde | 0.37 | | **0.62** | T2B, T3B; T3G |
| | (3β)-Lup-20(29)-en-28-al | 0.29 | | 0.23 | |
| | Betulinic acid | 0.53 | | **0.66** | T1B÷T3B; T1G, T3G |
| | Betulinaldehyde | **0.93** | T1B÷T3B; T1G÷T3G | 0.27 | |
| | Neurosporaxanthin methyl ester | **0.86** | T2B, T3B; T1G÷T3G | 0.31 | |
| Miscellaneous | Glycerol | **-0.61** | | 0.05 | |
| Unidentified compounds | Unidentified compound 1 | 0.50 | | **-0.59** | |
| | Unidentified compound 2 | -0.47 | | 0.15 | |
| | Unidentified compound 3 | **-0.89** | | 0.28 | |
| | Unidentified compound 4 | -0.47 | | **0.51** | |
| | Unidentified compound 5 | 0.20 | | 0.41 | |
| | Unidentified compound 6 | **0.79** | | 0.46 | |
| | Unidentified compound 7 | **0.67** | | 0.45 | |
| | Unidentified compound 8 | **0.67** | | 0.59 | |
| | Unidentified compound 9 | **0.73** | | 0.45 | |

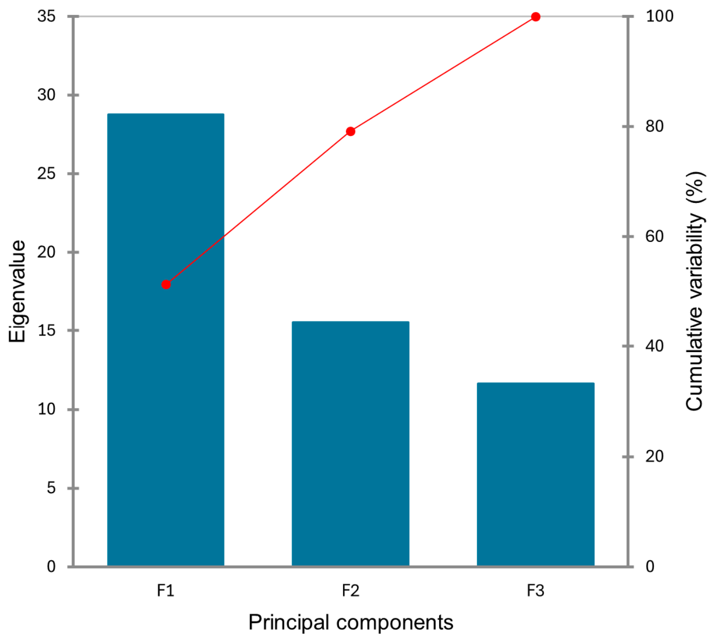


**Figure S3.** Scree plot of principal components showing explained variance for compounds in MeOH extracts across samples of leaves and galls induced by *Paracletus*, *Baizongia*, and *Geoica* aphids.

**Table S7.** Summary of principal components, eigenvalues, and variance explained for compounds across samples (MeOH extracts).

| | **F1** | **F2** | **F3** |
|---|---|---|---|
| Eigenvalue | 28.77 | 15.57 | 11.66 |
| Variability (%) | 51.38 | 27.80 | 20.82 |
| Cumulative % | 51.38 | 79.18 | 100 |

**Table S8.** PCA Factor loadings highlighting variation in median TIC% of individual compounds between leaves and galls of *Paracletus*, *Baizongia* and *Geoica* (MeOH extracts). Values in bold correspond to the factor for which the squared cosine is the largest.

| Compound class | Compound | F1 | Sample association | F2 | Sample association | F3 | Sample association |
|---|---|---|---|---|---|---|---|
| Hydrocarbons | *n*-Heptadecane | 0.47 | | **-0.85** | *Paracletus* | -0.25 | |
| | *n*-Octadecane | **0.86** | *Paracletus/Baizongia* | -0.50 | | 0.06 | |
| | *n*-Eicosane | -0.58 | | -0.41 | | **0.70** | *Baizongia* |
| Lipids | 1-Dodecanol | **0.68** | Leaves/*Paracletus* | 0.58 | | -0.45 | |
| | 1-Heptadecanol | **0.86** | *Paracletus* | -0.50 | | 0.06 | |
| | Methyl heptanoate | **0.80** | Leaves/*Paracletus* | -0.11 | | 0.60 | |
| | Palmitic acid | -0.24 | | **-0.95** | *Geoica* | -0.18 | |
| | 2-Monopalmitin | -0.06 | | **1.00** | Leaves | 0.08 | |
| | Methyl linolenate | **-0.99** | *Baizongia/Geoica* | -0.16 | | -0.05 | |
| | Methyl stearate | **0.71** | *Paracletus* | 0.69 | | -0.15 | |
| | 1-Monostearin | 0.24 | | **0.95** | Leaves | 0.18 | |
| Carbohydrates | Methyl β-D-ribofuranoside | **0.86** | *Paracletus* | -0.50 | | 0.06 | |
| | Methyl xylopyranoside 1 | **0.99** | Leaves/*Paracletus* | 0.16 | | 0.05 | |
| | Methyl xylopyranoside 2 | **0.68** | Leaves/*Paracletus* | 0.58 | | -0.45 | |
| | *L*-Rhamnose | **0.97** | *Paracletus* | 0.02 | | -0.23 | |
| | Ribose | **0.86** | *Paracletus* | -0.50 | | 0.06 | |
| | Pentonic acid-1,4-lactone | **0.68** | Leaves/*Paracletus* | 0.58 | | -0.45 | |
| | Ribonic acid | **-0.71** | *Baizongia* | -0.69 | | 0.15 | |
| | 2-Keto-*L*-gluconic acid | -0.26 | | **0.72** | *Baizongia* | 0.65 | |
| | Fructose | -0.34 | | 0.18 | | **0.92** | *Geoica* |
| | Galactose | **0.80** | Leaves/*Paracletus/Baizongia* | -0.11 | | 0.60 | |
| | Methyl galactoside isomer 1 | 0.33 | | 0.16 | | **0.93** | *Baizongia* |
| | Methyl galactoside isomer 2 | **0.99** | Leaves/*Paracletus* | 0.16 | | 0.05 | |
| | Galactose isomer | -0.34 | | 0.18 | | **0.92** | *Baizongia* |
| | Glucose isomer | **0.67** | *Paracletus* | -0.40 | | 0.62 | |
| | Galactitol | **0.97** | Leaves/*Paracletus* | 0.02 | | -0.23 | |
| | Methyl glucoside isomer 1 | **-0.67** | *Geoica* | -0.54 | | -0.50 | |
| | Methyl glucoside isomer 2 | -0.01 | | 0.39 | | **-0.92** | Leaves/*Paracletus* |

| | | | | | | | |
|---|---|---|---|---|---|---|---|
| | 4-*O*-Methyl-myo-inositol | -0.05 | | -0.69 | | **-0.73** | Geoica/*Paracletus* |
| | Galactonic acid | **0.80** | Leaves/*Paracletus* | -0.11 | | 0.60 | |
| | Gluconic acid | 0.24 | | **0.95** | *Paracletus* | 0.18 | |
| | Myo-Inositol | **0.80** | *Paracletus* | -0.11 | | 0.60 | |
| | 3-Deoxy-arabino-hexaric acid | **0.99** | Leaves/*Paracletus* | 0.16 | | 0.05 | |
| | Glucose 6-phosphate | **0.86** | *Paracletus* | -0.50 | | 0.06 | |
| | 2-*O*-Glycerol-$\alpha$-D-galactopyranoside | **0.86** | *Paracletus* | -0.50 | | 0.06 | |
| | Sucrose isomer | 0.33 | | 0.16 | | **0.93** | *Baizongia* |
| | Sucrose isomer | **0.71** | Leaves/*Paracletus* | 0.69 | | -0.15 | |
| | Disaccharide 2 | -0.54 | | **0.67** | Leaves/*Baizongia* | 0.51 | |
| | β-D-Lactose isomer 1 | **0.86** | *Paracletus* | -0.50 | | 0.06 | |
| | β-D-Lactose isomer 2 | **0.86** | *Paracletus* | -0.50 | | 0.06 | |
| | Disaccharide 9 | **0.86** | *Paracletus* | -0.50 | | 0.06 | |
| | Melibiose isomer 1 | -0.58 | | -0.41 | | **0.70** | *Baizongia*/*Geoica* |
| | Melibiose isomer 2 | **-0.71** | *Baizongia*/*Geoica* | -0.69 | | 0.15 | |
| Organic acids | Malic acid | 0.47 | | **-0.85** | *Paracletus* | -0.25 | |
| | Dimethyl malate | **0.86** | Missing in *Geoica* | -0.50 | | 0.06 | |
| | Shikimic acid | **-0.99** | *Baizongia*/*Geoica* | -0.16 | | -0.05 | |
| | (-)-Quinic acid | **0.71** | Leaves/*Paracletus* | 0.69 | | -0.15 | |
| | *DL*-2-Methylcitric acid | **0.99** | Leaves/*Paracletus* | 0.16 | | 0.05 | |
| Phenolics | Pyrogallol | 0.35 | | **-0.94** | *Paracletus* | 0.03 | |
| | Gallic acid | **0.97** | Leaves/*Paracletus* | 0.02 | | -0.23 | |
| | *trans*-*p*-Coumaric acid | **0.99** | Leaves/*Paracletus*/*Baizongia* | 0.16 | | 0.05 | |
| | 5-*O*-Coumaroyl-D-quinic acid | -0.58 | | -0.41 | | **0.70** | Baizongia/Geoica |
| | Caffeic acid | -0.34 | | 0.18 | | **0.92** | *Baizongia* |
| | Coniferylalcohol | 0.48 | | **-0.66** | *Paracletus* | 0.59 | |
| | Glycerol | **0.99** | *Paracletus* | 0.16 | | 0.05 | |

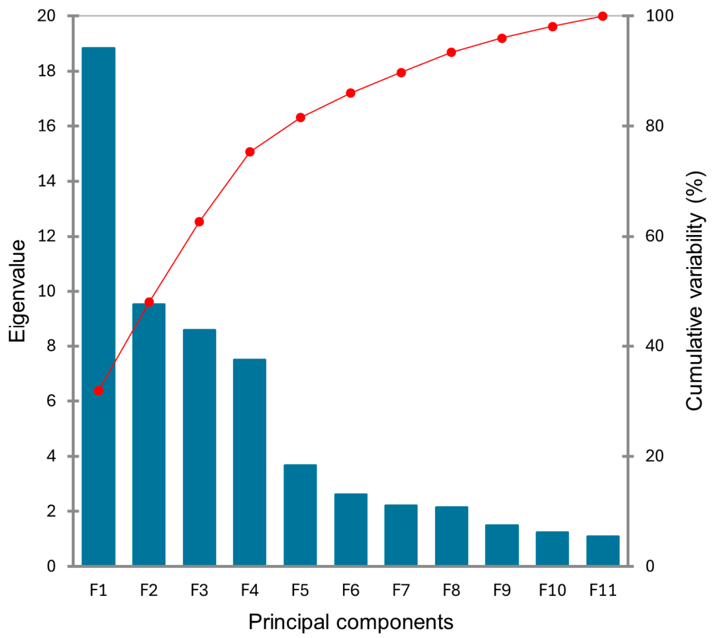


**Figure S4.** Scree plot of principal components and explained variance for compounds in MeOH extracts of leaves and galls induced by *Paracletus*, *Baizongia*, and *Geoica* aphids across trees.

**Table S9.** Summary of principal components, eigenvalues, and variance contributions for compounds across trees (MeOH extracts).

| | F1 | F2 | F3 | F4 | F5 | F6 | F7 | F8 | F9 | F10 | F11 |
|---|---|---|---|---|---|---|---|---|---|---|---|
| Eigenvalue | 18.84 | 9.55 | 8.56 | 7.50 | 3.68 | 2.61 | 2.22 | 2.16 | 1.51 | 1.26 | 1.10 |
| Variability (%) | 31.93 | 16.18 | 14.54 | 12.72 | 6.23 | 4.43 | 3.76 | 3.67 | 2.55 | 2.13 | 1.86 |
| Cumulative % | 31.93 | 48.12 | 62.65 | 75.36 | 81.60 | 86.03 | 89.79 | 93.46 | 96.01 | 98.14 | 100.00 |

**Table S10**. PCA factor loadings highlighting variation in raw TIC% of individual compounds in MeOH extracts of leaves and galls induced by *Paracletus*, *Baizongia*, and *Geoica* aphids across individual trees (T1, T2, T3). Values in bold correspond to the factor for which the squared cosine is the largest.

| Compound class | Compound | F1 | Tree association | F2 | Tree association | F3 | Tree association |
|---|---|---|---|---|---|---|---|
| Hydrocarbons | *n*-Octadecane | **0.52** | **T2** | -0.32 | | 0.08 | |
| | *n*-Eicosane | 0.03 | | **0.87** | **T1** | 0.22 | |
| Lipids | 1-Dodecanol | **0.71** | **T2** | -0.53 | | -0.04 | |
| | Methyl heptanoate | **0.86** | **T1** | 0.34 | | 0.10 | |
| | Palmitic acid | -0.02 | | **0.51** | **T1** | -0.32 | |
| | Methyl linolenate | **-0.76** | **T3** | -0.16 | | 0.13 | |
| | Methyl stearate | **0.68** | **T1** | 0.23 | | -0.41 | |
| Carbohydrate | 2-Keto-*L*-gluconic acid | -0.21 | | 0.26 | | **0.72** | **T2** |
| | Fructose isomer 1 | 0.08 | | 0.17 | | **0.90** | **T2** |
| | Galactose isomer 1 | 0.38 | | -0.22 | | **0.79** | **T2** |
| | Methyl galactoside isomer 1 | 0.13 | | 0.01 | | **0.92** | **T2** |
| | Methyl galactoside isomer 1 | **0.86** | **T1,T3** | -0.16 | | -0.17 | |
| | Fructose isomer 2 | **0.53** | **T2** | 0.43 | | 0.01 | |
| | Galactose isomer 2 | 0.22 | | **0.61** | **T2** | 0.51 | |
| | Glucose isomer | 0.56 | | -0.01 | | **0.59** | **T2** |
| | Galactitol | **0.91** | **T2, T3** | -0.14 | | -0.31 | |
| | Methyl glucoside isomer 1 | -0.48 | | **0.60** | **T1,T3** | -0.13 | |
| | Methyl glucoside isomer 2 | 0.24 | | **-0.56** | **T2** | -0.37 | |
| | 4-*O*-Methyl-myo-inositol | -0.47 | | -0.09 | | **-0.73** | **T3** |
| | Methyl β-D-ribofuranoside | **0.63** | **T2, T3** | 0.06 | | -0.35 | |
| | Methyl xylopyranoside isomer 1 | **0.81** | **T3** | -0.35 | | -0.12 | |
| | Methyl xylopyranoside isomer 2 | **0.72** | **T1, T2, T3** | -0.53 | | -0.26 | |
| | *L*-Rhamnose | **0.64** | **T2** | -0.10 | | 0.45 | |
| | Ribose | **0.59** | **T2** | -0.42 | | 0.19 | |
| | Ribonic acid | -0.36 | | **0.60** | **T3** | -0.14 | |
| | Myo-Inositol | 0.54 | | -0.34 | | **0.61** | **T2** |
| | 3-Deoxy-arabino-hexaric acid | **0.89** | **T1** | -0.10 | | -0.15 | |

| | | | | | | | |
|---|---|---|---|---|---|---|---|
| | Glucose 6-phosphate | **0.72** | **T1** | 0.41 | | -0.24 | |
| | 2-O-Glycerol-α-D-galactopyranoside | **0.72** | **T1** | 0.46 | | -0.29 | |
| | Sucrose isomer 1 | 0.46 | | 0.47 | | **0.64** | **T2** |
| | Sucrose isomer 2 | **0.87** | **T1** | 0.12 | | -0.07 | |
| | Disaccharide 3 | **0.56** | **T1** | 0.42 | | -0.06 | |
| | β-D-Lactose isomer 1 | **0.73** | **T1** | 0.45 | | -0.31 | |
| | β-D-Lactose isomer 2 | **0.83** | **T1** | 0.29 | | -0.28 | |
| | Disaccharide 6 | **0.56** | **T1** | 0.47 | | -0.24 | |
| | Disaccharide 9 | **0.76** | **T1** | 0.47 | | -0.15 | |
| | Melibiose isomer 1 | 0.04 | | **0.81** | **T1** | -0.10 | |
| | Melibiose isomer 2 | **-0.72** | **T1** | 0.54 | | -0.02 | |
| | Galactonic acid | 0.54 | | -0.34 | | **0.61** | **T2** |
| Organic acids | Dimethyl malate | **0.55** | **T2** | 0.35 | | 0.07 | |
| | Shikimic acid | **-0.62** | **T3** | 0.39 | | 0.07 | |
| | (-)-Quinic acid | **0.63** | **T3** | -0.27 | | -0.50 | |
| | *DL*-2-Methylcitric acid | **0.65** | **T1, T3** | -0.04 | | -0.33 | |
| Phenolics | *trans*-*p*-Coumaric acid | **0.74** | **T1, T2** | 0.31 | | 0.09 | |
| | 5-*O*-Coumaroyl-D-quinic acid | 0.08 | | **0.86** | **T1** | 0.06 | |
| | Caffeic acid | 0.12 | | 0.05 | | **0.85** | **T2** |
| | Gallic acid | **0.76** | **T1, T2** | -0.26 | | 0.03 | |
| | Coniferylalcohol | **0.65** | **T1** | 0.54 | | 0.04 | |
| Miscellaneous | Glycerol | 0.16 | | 0.05 | | **-0.57** | **T3** |
| | Unidentified compound 3 | **-0.57** | | 0.47 | | 0.07 | |
